# Mapping the Genetic Basis of Symbiotic Variation in Legume-Rhizobium Interactions in *Medicago truncatula*

**DOI:** 10.1534/g3.112.003269

**Published:** 2012-11-01

**Authors:** Amanda J. Gorton, Katy D. Heath, Marie-Laure Pilet-Nayel, Alain Baranger, John R. Stinchcombe

**Affiliations:** * Department of Ecology and Evolutionary Biology, University of Toronto, Toronto, Ontario M5S 3B2, Canada; † Department of Plant Biology, University of Illinois at Urbana-Champaign, Urbana, Illinois, 61801; ‡ Amélioration des Plantes et Biotechnologies Végétales, INRA-Agrocampus-Ouest-Université de Rennes 1, 35653 Le Rheu Cedex, France

**Keywords:** G × G, genotype by genotype interactions, QTL mapping, Nod factor signaling, *Sinorhizobium meliloti*

## Abstract

Mutualisms are known to be genetically variable, where the genotypes differ in the fitness benefits they gain from the interaction. To date, little is known about the loci that underlie such genetic variation in fitness or whether the loci influencing fitness are partner specific, and depend on the genotype of the interaction partner. In the legume-rhizobium mutualism, one set of potential candidate genes that may influence the fitness benefits of the symbiosis are the plant genes involved in the initiation of the signaling pathway between the two partners. Here we performed quantitative trait loci (QTL) mapping in *Medicago truncatula* in two different rhizobium strain treatments to locate regions of the genome influencing plant traits, assess whether such regions are dependent on the genotype of the rhizobial mutualist (QTL × rhizobium strain), and evaluate the contribution of sequence variation at known symbiosis signaling genes. Two of the symbiotic signaling genes, *NFP* and *DMI3*, colocalized with two QTL affecting average fruit weight and leaf number, suggesting that natural variation in nodulation genes may potentially influence plant fitness. In both rhizobium strain treatments, there were QTL that influenced multiple traits, indicative of either tight linkage between loci or pleiotropy, including one QTL with opposing effects on growth and reproduction. There was no evidence for QTL × rhizobium strain or genotype × genotype interactions, suggesting either that such interactions are due to small-effect loci or that more genotype-genotype combinations need to be tested in future mapping studies.

Mutualisms, or interspecific cooperation, are reciprocally beneficial interactions between species (Bronstein 2001) that affect many ecological and evolutionary processes. Organisms from every kingdom participate in mutualisms, from mitochondria within cells, to mycorrhizal fungi and plants, to cleaner fish and their clients (Boucher 1985; Smith and Douglas 1987; Bronstein 1994). These mutualistic interactions are known to be genetically variable, where different genotypes confer different fitness benefits to their interacting partners [*e.g.* Hoeksema and Thompson (2007) and Heath (2010)]. A number of important unresolved questions remain, however, including the mechanistic basis of such genetic variation in fitness and whether the loci responsible for such fitness variation are dependent on the genotype of the interacting partner. The prevalence, distribution, and effect size of loci for fitness benefits, and their potential for partner-specificity, have important implications for the genetic basis of ongoing coevolution (Parker 1995; Gomulkiewicz *et al.* 2007) and how genetic architecture influences the maintenance of mutualisms (Bever 1999; Heath and Tiffin 2007; Heath 2010). Here we used quantitative trait locus mapping to investigate genomic regions that influence plant performance and fitness of the legume *Medicago truncatula*, whether those regions depend on the genotype of its associated rhizobial mutualist, and the contribution of variation in known mutualism signaling genes.

At the quantitative genetic level, genotype × genotype interactions (G × G) for fitness appear to be widespread in coevolutionary interactions (Moran 1981; Service 1984; Salvaudon *et al.* 2005; Hoeksema and Thompson 2007; Heath 2010). These data have two important implications. First, they suggest widespread genetic variation in fitness components of interacting species, indicating abundant raw material for evolutionary change in interspecific interactions, as the loci underlying fitness differences represent the genetic variation upon which coevolutionary selection acts (Parker 1995; Gomulkiewicz *et al.* 2007). Second, the presence of G × G interactions suggests that the loci responsible for fitness variation may be partner specific. These G × G interactions, also referred to as intergenomic epistasis (Wade 2007), occur when the effects of a gene in one partner vary depending on the genetic background of the other partner. Applied to mutualisms, they indicate that the phenotype of an individual can change depending on the genome of its interacting partner: a poor mutualist partner with one individual may be beneficial with another, potentially stabilizing mutualisms (Heath and Tiffin 2007; Heath 2010).

In legume-rhizobium symbioses, rhizobia fix atmospheric nitrogen into a plant-usable form in exchange for shelter inside specialized plant root nodules and plant photosynthates. Using numerous rhizobium strain × plant line combinations from natural populations of the model legume *Medicago truncatula* and its nitrogen-fixing rhizobium *Sinorhizobium meliloti*, Heath and colleagues detected G × G interactions for plant fitness components [N = 20 line × rhizobium strain combinations (Heath and Tiffin 2007); N = 108 line × rhizobium strain combinations (Heath 2010)] and further showed that some rhizobium strains were highly beneficial mutualists with certain plant genotypes but provided little or no benefit when paired with other plant genotypes. Given these past results, it is expected that significant rhizobium strain × quantitative trait loci interactions may exist. However, despite the variety of genetic and genomic resources available in *M. truncatula* [*e.g.* genome sequenced and assembled, euchromatin draft sequence available (Young *et al.* 2011), haplotype maps (Branca *et al.* 2011), gene expression atlas, available RIL mapping populations, and genetic maps], the causative loci or regions underlying quantitative genetic variation in plant fitness and their potential partner-specific effects remain unknown.

Signaling interactions between rhizobia and legumes are essential to the formation of the symbiosis and, as a result, are promising candidates to investigate whether sequence-level variation affects the outcome or fitness benefits of symbiotic interactions. Several genes involved in the signaling pathway between rhizobia and legumes have already been identified (Schauser *et al.* 1999; Catoira *et al.* 2000; Endre *et al.* 2002; Ané *et al.* 2004; Lévy *et al.* 2004; Arrighi *et al.* 2006). Briefly, nodule formation and host specificity are controlled by mutual signaling between the two partners. Legumes release flavonoids into the soil, and these molecules trigger rhizobia to produce Nod factors. Nod factors are lipochito-oligosaccharide compounds, which in turn induce multiple downstream responses in the host, ultimately leading to the formation of root nodules [see Jones *et al.* (2007) for a review]. In the last decade, five genes involved in the earliest stages of the Nod factor recognition and signaling pathway have been characterized in *M. truncatula*: *DMI1*, *DMI2*, *DMI3*, *NFP*, and *NIN* [see Figure 1 in De Mita *et al.* (2007) and Figure 2 in Jones *et al.* (2007) for diagrams of the signaling pathway]. *DMI1* encodes an ion channel located on the nuclear membrane (Ané *et al.* 2004); *DMI3* encodes a calcium-calmodulin–dependent kinase (Lévy *et al.* 2004); *NFP* is a lysine motif (LysM)-receptor-like kinase and is the candidate for the Nod factor receptor gene (Arrighi *et al.* 2006); *DMI2* encodes a receptor kinase and is involved in rhizobial Nod factor perception (Endre *et al.* 2002); and *NIN* is required for the formation of the plant infection threads and has homologies to transcription factors (Schauser *et al.* 1999). Mutants with nonfunctional copies of any of these genes cannot be infected by rhizobia and therefore produce no nodules (Schauser *et al.* 1999; Catoira *et al.* 2000; Amor *et al.* 2003).

Quantitative trait loci (QTL) mapping is an excellent method for locating regions of the genome affecting ecologically and evolutionarily important traits. Using a recombinant inbred line (RIL) mapping population of *M. truncatula*, we examined the QTL architecture of a range of plant fitness traits when grown with two genotypically distinct *S. meliloti* strains. Specifically, we addressed the following questions: (1) Which regions of the *M. truncatula* genome contribute to variation in plant fitness? (2) Do the number and effect size of additive and epistatic QTL differ in two rhizobium genotype treatments (*i.e.* are QTL differentially detected depending on the rhizobium strain)? and (3) Does sequence level variation in known symbiosis genes have an effect on the ecological outcome of the symbiosis?

## Materials and Methods

### Study species and mapping population

*Medicago truncatula* (Fabaceae), commonly known as the barrel medic, is a small annual legume native to the Mediterranean region. *Medicago truncatula* has trifoliate leaves and small inflorescences with one to five yellow flowers; it is found mainly in open areas (Bataillon and Ronfort 2006). It has been developed as the model legume for studying the genetics of plant-mycorrhizal and plant-rhizobium symbioses due to its short generation time, high selfing rate [∼97.5% (Bonnin *et al.* 1996)], and small diploid genome [2n = 16; 550 Mb (Cook 1999; Ané *et al.* 2008; Young *et al.* 2005)]. *Medicago truncatula* is found in symbiosis with the nitrogen-fixing bacteria *S. medicae* and *S. meliloti* (Rhizobiaceae) (Bailly *et al.* 2006). In our experiment, we used *S. meliloti*.

Seeds from the *M. truncatula* LR03 RIL mapping population were provided by INRA in Montpellier, France. The lines were created by manually crossing two *M. truncatula* inbred line accessions, F803005.5 (female parent, origin: France) and DZA045.5 (male parent, origin: Algeria) to produce an F_1_ generation, which was later selfed. The RILs (n = 177) were derived from the F_2_ generation by self-fertilization and single-seed descent for five generations, creating the F_6_ RIL mapping population that we used.

Both *S. meliloti* strains used in this experiment (*Naut a* and *Sals b*, hereafter referred to as *Naut* and *Sals*) are from the native range in France and were previously isolated and genotyped from soil samples [full details in Heath (2010)]. *Naut* and *Sals* were selected based on preliminary results indicating evidence of G × G interactions with the parental lines of the LR03 RIL population (ANOVA for leaf number, rhizobium strain × parental line, *P* = 0.02, supporting information, Figure S1). The presence of G × G interactions suggested that *Naut* and *Sals* would be suitable strains to investigate possible rhizobium strain × QTL interactions in our experiment.

### Greenhouse experiment

#### Planting design:

We grew 8 replicates of each RIL with each of two rhizobium strain treatments. To minimize contamination, rhizobium treatments were applied at the level of whole groups of plants (*i.e.* on a bin-by-bin basis). Specifically, one seedling per line was planted into Ray-Leach SC10 UV-stabilized cone-tainers (Stuewe and Sons, Tangent, OR); we then placed four racks of cone-tainers into Flow Trays (large plastic “bins” that provide subirrigation), each of which received its own rhizobium strain inoculation. Within each bin, there were 177 genotyped RILs, 4 replicates of each of the parental lines, and an additional 10 RILs that lacked genetic markers, which were included to maintain an even plant density across bins (N = 195 plants per bin). We used 8 replicate bins per rhizobium treatment for a total of 16 bins, arranged in the greenhouse in a checkerboard array of 8 blocks (one bin of each strain per block) to eliminate any potential confounding relationships between rhizobium treatment and spatial position (195 plants × 8 replicates × 2 treatments = 3120 experimental plants). Bins were approximately ten inches apart on the greenhouse bench. To assess the potential for cross-contamination in our experiment, a tray of 50 uninoculated control plants was placed adjacent to inoculated trays (with between-tray spacing and watering regime identical to the rest of the experiment). Plant mortality, growth, and nodule data for these controls suggest that cross-contamination was minimal (see *Results*).

All seeds were scarified with a razor blade, then surface sterilized for 30 sec in 95% ethanol, followed by 7 min in full bleach. Following sterilization, seeds were imbibed in ddH_2_0 for 20 min, plated on 0.08% agar plates, and stratified in the dark at 4° for 2 weeks. After 2 weeks, the seedlings were transplanted into 164 mL cone-tainers containing a mixture of 1:1 Sunshine Mix 2 (Sun Gro Horticulture, Bellevue, WA) and Turface (Profile Products, Buffalo Grove, IL). We used Sunshine Mix 2 because it contains no added fertilizer, as it has been shown previously that nitrogen can affect the outcome of the symbiosis (Heath and Tiffin 2007; Heath *et al.* 2010). Prior to transplanting, the soil mixture was steam sterilized at 121° for 30 min in small bags. We planted one seed per line in a randomized design within each bin, and cone-tainers were placed in every other space in each 98-cell rack. Planting was completed on a bin-by-bin basis from Jan. 1 through Jan. 6, 2010. Seeds that failed to establish were replaced with a second set of seedlings a month later using the same methods described above. We fertilized all plants once with N-free Fahreus solution (Somasegaran and Hoben 1994) and grew the plants under 16-hr days at an average greenhouse temperature of 22°.

#### Rhizobium strain inoculation:

One week after planting, we inoculated all plants with the rhizobium cultures. To prepare rhizobium inocula, each strain was first grown in liquid culture [TY media; Beringer (1974)] for three days at 30° and mixed continuously at a rate of 200 rpm. Prior to inoculation, we diluted each culture to a density of 0.1 OD_600_. We then inoculated each plant with 1 mL of the appropriate rhizobium strain (*Sals* or *Naut*). The following day, we poured 25 mL of sterile water on the soil of each cone-tainer to help distribute the rhizobium cells throughout the soil.

#### Trait measurement:

For each plant, we recorded seven measures of plant growth, size, or fitness: leaf number at six weeks, date of first flower, number of fruits, fruit weight, dry shoot weight, dry root weight, and number of primary branches. We used fruit weight and fruit number as estimates of reproductive fitness, and both are positively correlated with seed number [fruit weight and seed number: *r* = 0.75, *P* < 0.0001 (A. J. Gorton, unpublished data); fruit number and seed number: *r* = 0.86, *P* < 0.0001 (Heath and Tiffin 2007)]. For leaf number, we counted the number of true leaves present on each plant at six weeks after planting as a measure of plant growth. We checked all plants daily for signs of flowering. Once the plants began to set fruit, we collected mature fruit daily.

The plants were harvested in sequential order based on planting date approximately 15 weeks after the first day of planting. Prior to harvesting, all remaining fruit was removed to estimate total fruit number and fruit weight. At harvest, we separated aboveground and belowground biomass, which were then dried at 55° for three days prior to weighing. Average fruit weight was calculated as the weight of all fruits for each plant divided by the total number of fruits. After harvesting, dried shoots were counted to assess the number of primary branches on each plant [See Moreau *et al.* (2006) for a description of branching; for complete phenotypic data, see File S1.]

During harvesting, we observed nodules on the roots of all experimental plants; however, the roots were very dense and contained numerous nodules, making it difficult to collect data on nodule traits on all experimental plants in a timely manner (*i.e.* before roots began to degrade). Accordingly, we subsampled nodules in the following manner: all nodules on every parental genotype were counted, and a subset of 15–20 nodules was randomly selected from each parental genotype for weighing. An analysis of the parental lines indicated that there was no effect of rhizobium strain on nodule number (*Naut* = 129.9; *Sals* = 111.9; *P* = 0.54) or dry weight (*Naut* = 0.027; *Sals* = 0.019; *P* = 0.65), nor was there a strain × line interaction for either trait (*P* = 0.32, *P* = 0.34).

### Candidate gene sequencing

To test for sequence variation at particular genes involved in the Nod factor signaling pathway (*DMI1*, *DMI2*, *DMI3*, *NIN*, and *NFP)*, the parental lines and RILs were initially sequenced using DNA derived from a separate planting set. DNA was extracted from leaf tissue according to DNAeasy Plant Mini Kits protocol (Qiagen). *DMI1* primers were taken from De Mita *et al.* (2007). The remaining primer pairs were designed using Integrated DNA Technologies (IDT) PrimerQuest (www.idtdna.com/Scitools/Applications/Primerquest), and BLASTn was used to ensure they matched only the gene of interest in the *M. truncatula* genome (see Table S1 for primer sequences).

Purified PCR products were sequenced with the forward primers using Big Dye Terminator cycle sequencing kit (v.3.1, Applied Biosystems), which were then run on an ABI 3730 DNA Analyzer by the Centre for the Analysis of Genome Evolution and Function (University of Toronto). The parental lines and RILs were sequenced twice, and we aligned the sequences for each gene using ClustalW in BioEdit (Hall 1999).

Only *DMI1*, *DMI3*, and *NFP* differed between the parents; therefore, they were fully sequenced in the RILs to develop markers for QTL mapping. In *DMI1* (5815 bp), a fifth of the gene was successfully sequenced, and the fragment contained three polymorphisms: one was a nonsynonymous change resulting in the substitution of isoleucine for threonine, and two were in introns. The nonsynonymous SNP found in the *DMI1* fragment was used to genotype the RILs (position in gene = 388 bp). The entire *NFP* gene (1788 bp) was sequenced, and three polymorphisms were found, two of which were synonymous changes and one of which was a nonsynonymous change, resulting in the substitution of tryptophan for serine in the protein sequence. One of the synonymous changes in *NFP* was used to genotype the RILs (position in gene = 792 bp). In *DMI3* (6700 bp), a fifth of the gene was successfully sequenced, and this segment contained five polymorphisms within introns; the SNP at position 1921 bp in *DMI3* was used to genotype the RILs (see Table S2 for details on SNPs used for genotyping; see File S1 for genotype scores).

### Statistical analyses

#### Quantitative genetic analysis:

We tested for genetic variation in plant traits in separate analyses for each rhizobium strain treatment by using a mixed-model ANOVA with restricted maximum likelihood (PROC MIXED, SAS v.9.2). For each trait as a response variable, we included block and planting set as fixed effects and line as a random effect. The significance of the line effect was tested by running the models with and without the line effect; the difference in the −2 log likelihoods of the models was compared to chi-square distribution with 1 degree of freedom, using a one-tailed test. To formally test for line × rhizobium genotype interactions, we also used mixed-model ANOVA. For each trait as a response variable, we included block, planting set, and rhizobium genotype as fixed effects, and line and line × rhizobium genotype as random effects. Significance tests were performed as described above. We report broad sense heritability (H^2^) estimates as the genetic variance (V_g_) divided by the phenotypic variance (V_p_, where V_p_ = V_g_ + V_e_), so that our estimates would be comparable with many past reports [*e.g.* Hansen *et al.* (2011)]; these REML estimates are robust to lack of balance among RILs (Lynch and Walsh 1998). Correlations between phenotypic traits were calculated using least-square RIL means (PROC CORR, SAS v.9.2).

As a point of comparison, we also estimated heritability on the line-mean basis, using V_g_ / [V_g_ + (V_e_ / n-reps)], which is often used in the breeding literature when the phenotype of interest is the mean of a line [*e.g.* Holland *et al.* (2003), Long *et al.* (2006), Piepho and Mohring (2007), and Emrich *et al.* (2008)]. The equation for the line-mean heritability only provides an accurate predictor on the response to selection of line means under conditions of equal balance (Piepho and Mohring 2007), which was not met in the current experiment. Because of the lack of balance, for the number of replicates per line we used the *n_0_*, an estimate of the weighted RIL sample size, calculated with expressions from Lynch and Walsh (1998). Thus, while the heritability on the line-mean basis we report should not be used for predicting response to selection of line-mean phenotypes, it provides a measure of the degree of variability among RIL means, which were used for QTL mapping.

#### Linkage map construction:

The RILs were genotyped at 204 markers, of which most are simple sequence repeats (SSR) and amplified fragment length polymorphisms (AFLP). AFLP assays were conducted using *EcoRI* and *MseI* restriction enzymes, and SSR amplifications were carried out using the procedure described on pea by Loridon *et al.* (2005), with fluorescently labeled (IRD 700 or IRD 800) forward primers (see File S2, Table S6, and Table S7 for more details on AFLP and SSR amplification methods). The remaining three markers (DMI1441, NFP1697, and DMI3427) are single nucleotide polymorphisms which were designed from sequence data from the symbiotic signaling pathway genes *DMI1*, *NFP*, and *DMI3* using the method described above. The markers that are anchored to the integrated genetic-physical *M. truncatula* map (http://www.medicago.org/genome/map.php) are indicated in bold on the linkage map (Mun *et al.* 2006; Hamon *et al.* 2010; this study).

We used JoinMap 4.0 (Van Ooijen 2006) to determine the linkage map of the population (N= 204 markers). We determined the linkage groups using a LOD score between 4 and 8, and the marker order and distances between were calculated using maximum-likelihood mapping. For some of the linkage groups, multiple marker orders were generated. In these cases, we investigated the raw genotype data for evidence of improbable double recombinants or transcript reading errors. We excluded a small number of markers from the analysis for these reasons, if they were located in regions of reasonable marker density (*i.e.* ∼8–10 cM between markers).

#### QTL analysis:

We performed QTL mapping separately for each strain treatment. The explanation for this approach is that strain-specific QTL may exhibit conditional neutrality [*sensu* (Mackay 2001], in which they affect phenotypes in one strain treatment but have no effect in the other. If the QTL that display conditional neutrality differ between the two rhizobium treatments, it is possible for different QTL to affect traits in the different strain treatments, even in the absence of a significant line × rhizobium strain treatment interaction.

For the QTL mapping, we estimated the least-squares means of each plant trait using a mixed-model ANOVA (PROC MIXED, SAS v.9.2), which included block, plant set, and line as fixed effects. We did these estimations and all QTL mapping analyses described below separately for each rhizobium strain treatment. We did not estimate best linear unbiased predictors (BLUP) or the random effects solutions of the models described above, because BLUPs can have poor properties when used as data in regression-based analyses (Hadfield *et al.* 2010).

QTL were initially detected using the composite interval mapping (CIM) procedure (Jansen 1993; Zeng 1993, 1994) in QTL Cartographer v.2.5 (Wang *et al.* 2011). The cofactors used in each CIM were selected using forward-backward stepwise regression (*P*-values = 0.05) under the standard model (Model 6). All QTL analyses were performed on the calculated least-squares means of each RIL, and tests were performed at 2 cM intervals with a window size of 10 cM. For each trait, the genome-wide threshold values [likelihood ratio test statistic (LRT); *P* = 0.05] for declaring a significant QTL were determined through 1000 permutations of the data set (Churchill and Doerge 1994). For significant QTL, we calculated 2-LOD support intervals as the nearest markers on either side of the QTL peak where the LRT dropped by 9.22 (Van Ooijen 1992).

Many of the traits had QTL that colocalized within each strain treatment. To formally test for pleiotropy at the QTL level, we implemented multiple trait CIM (MCIM) in QTL Cartographer v.2.5 (Wang *et al.* 2011). Multiple trait CIM takes into account the correlated structure of phenotypic traits to map QTL affecting multiple traits (Jiang and Zeng 1995). The analysis was performed only on those QTL identified under single-trait CIM that had overlapping 2-LOD intervals. The LRT values for declaring a significant QTL (using a 0.05 type I error rate) were determined through 1000 permutations of the data set, maintaining the correlations between traits (Churchill and Doerge 1994). For each position where MCIM detected a significant QTL, we examined the individual MCIM LRT values for each trait to determine whether the detected QTL had pleiotropic effects on the traits in each analysis. QTL are referred to as “pleiotropic” and share the same QTL identification (see *Results*) when more than one trait had a LRT value greater than the significance threshold of 5.99 [*χ*_0.05, 2_; Jiang and Zeng (1995)] at the detected joint QTL position. QTL are referred to as “closely linked” and were given unique identifications when only one trait in the MCIM analyses was significant for the detected QTL.

We performed the final model fit using multiple interval mapping [MIM; Kao *et al.* (1999)] in QTL Cartographer v.2.5 (Wang *et al.* 2011). MIM uses multiple marker intervals concurrently to fit several potential QTL in the model, thereby increasing power and precision (Kao *et al.* 1999). For each trait, we used our CIM QTL peaks as the initial positions in MIM, tested to determine whether all remained in the model simultaneously, and omitted any that did not. We used BIC–M2 = 2ln(n) as our criteria for keeping QTL in the model. QTL effects and percentage variance explained were estimated with a final model fit in MIM, as CIM does not account for correlations among genotypes due to sampling in the RIL population and therefore can overestimate these values. All significant QTL were drawn on the linkage map using MapChart (Voorrips 2002).

To determine whether QTL were differentially detected depending on the rhizobium genotype (*i.e.* QTL × strain treatment or QTL × E), we performed a multiway ANOVA for each trait (PROC GLM, SAS v.9.2). We selected the markers closest to the peak of each significant QTL in the two strain treatments, and we included all of these markers, as well as two-way marker × rhizobium strain interactions, as main effects in the model. A formal statistical test is needed because it is possible for a QTL to have a significant phenotypic effect in one environment but have little or no effect in the other environment and thus remain undetected due to low statistical power.

We also tested for epistatic interactions between markers (epistatic QTL) for each trait in both strain treatments using the program Epistacy (Holland 1998). Epistacy tests for the effect of all possible pairwise combinations of the markers on each trait using SAS (PROC GLM), regardless of whether a significant additive QTL was detected. To account for the problem of multiple testing and determine which results were truly significant, we implemented a false discovery rate (FDR) approach using QVALUE (Storey and Tibshirani 2003) with FDR set at 0.05. For each test of significance, QVALUE assigns a *q*-value to each *P*-value, indicating the probability that the result in question is a false discovery, given that it is interpreted as statistically significant. Only those *P*-values that survived FDR are reported. For significant epistatic interactions that were unlikely to be false-positives (*i.e. P* < 0.05, *q* < 0.05), we also tested for QTL × QTL × E by including the two significant markers, the two-way marker × marker interaction, and the three-way marker × marker × rhizobium strain interaction.

In addition to the strain-specific QTL mapping, we mapped QTL using phenotypes estimated as the line mean squares across both strain treatments. We estimated line means using a model that included block, strain, plant set, and line as fixed effects, and line × strain, strain × block, and line × strain × block as the random effects. The last two random effects were included because the rhizobium treatments were applied to half a block at a time, rather than to individual plants.

#### Candidate gene and QTL colocalization analysis:

We evaluated the chance that our Nod factor signaling candidate genes could underlie QTL in two ways. First, we used a randomization test to evaluate the possibility that any QTL would overlap a candidate gene. To do so, we randomly selected markers as positions in the genome and randomly assigned QTL 2-LOD intervals in cM to them, centered on the marker; the number of markers selected corresponded to the number of QTL detected in that experimental treatment, and the 2-LOD intervals used corresponded to the cM 2-LOD intervals of all QTL detected in that experimental treatment. We repeated this analysis 10,000 times and calculated the number of cases where a randomly assigned QTL interval overlapped one of our candidate genes. The probability that a QTL will have a 2-LOD interval overlapping a candidate increases with the number of traits measured, QTL detected, and candidate genes placed on the map. Second, we tested trait and gene-specific hypotheses, repeating the analysis above, but using only the number of QTL and the marker intervals for the actual traits that mapped to candidate genes. These analyses test the hypothesis that a QTL for fruit weight, for example, will overlap the detected candidate gene by chance alone, given the number of fruit weight QTL detected and their associated confidence intervals.

## Results

### Survival and control plants

In total, 2078 plants survived, with a mean and median of 5 plants per line in each strain treatment, which has been shown to be sufficient for QTL mapping (Keurentjes *et al.* 2007). Our control tray suggested that cross-contamination in the greenhouse was minimal. Only 1 control plant (out of 50) formed nodules; moreover, that plant formed only one large nodule, suggestive of a single contaminating cell, whereas a sample of inoculated plants indicated that mean nodule number was 108.7 (n = 128, SE ± 6.96). Out of the remaining 49 control plants, only 29 survived, and these plants were small, chlorotic, and did not flower or set seed. Although minimal, the single inoculated control plants suggest that cross-contamination was possible in our experiment; nevertheless, any contamination would be unlikely to bias our results for multiple reasons. First, the large populations (10^6^) of rhizobium cells added to inoculated plants should swamp the effects of contaminating strains. Moreover, because the placement of each plant genotype was randomized within each bin, any among-bin contamination should be random with respect to plant genotype and thus should not bias estimates of rhizobium strain effects in our analyses. Finally the significant differences in belowground biomass of plants with *Naut vs. Sals* (*P* = 0.0076) suggests that rhizobium strain treatments were in fact distinct.

### Quantitative genetics

**Figure 1:**
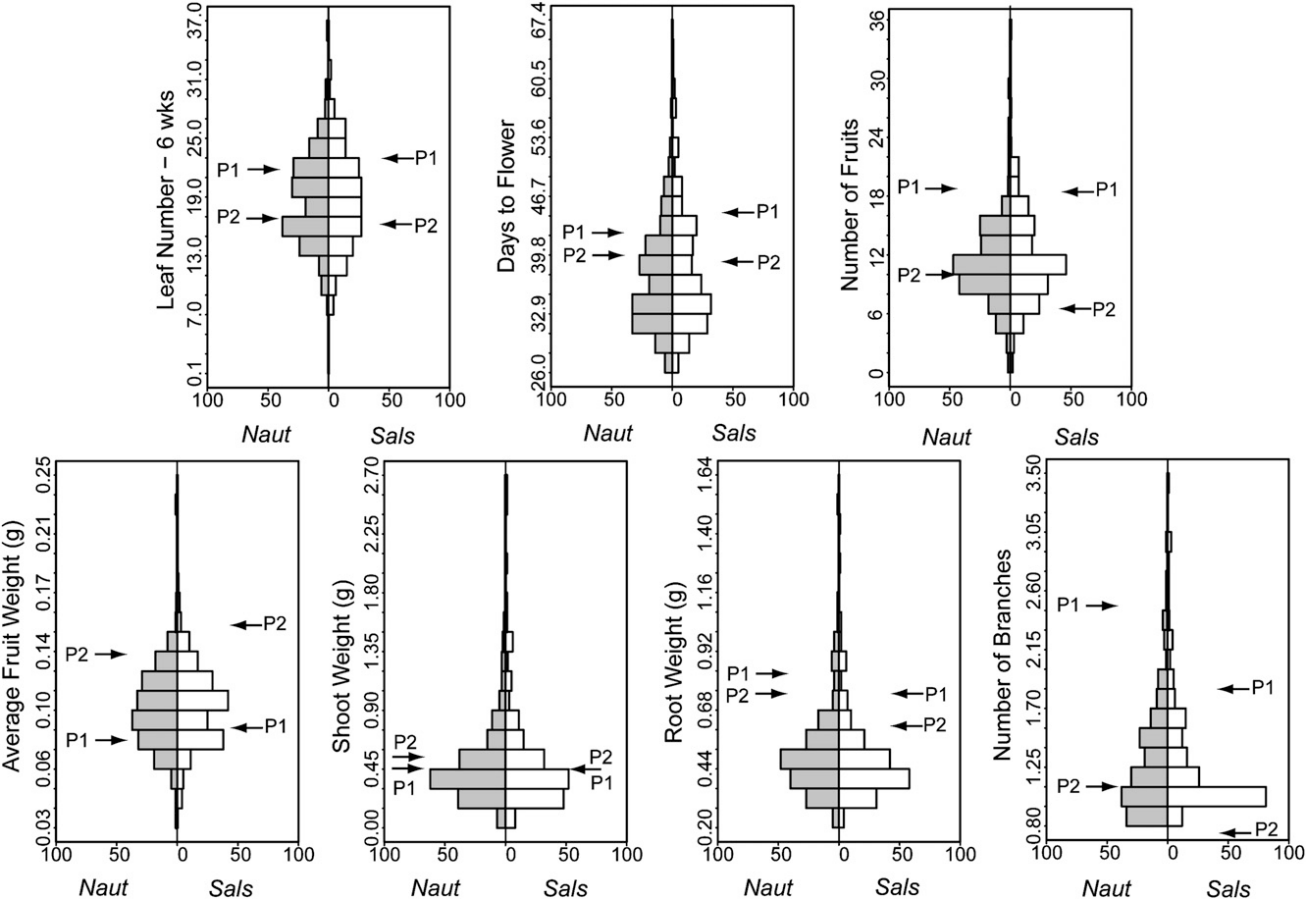
Back-to-back histograms of trait least-square means of the *M. truncatula* RILs grown with rhizobium strains *Naut* (gray bars) and *Sals* (white bars). Parental trait means are indicated by arrows (P1 = female parent, line F83005.5; P2 = male parent, line DZA045.5).

We detected significant genetic variation in all phenotypic traits in both rhizobium strain treatments (Table 1). Contrary to our preliminary results, we did not find any significant line × rhizobium strain interaction in either strain treatment (*P* = 0.5 for all traits), and a comparison of the RIL trait least-squares means when grown with *Naut* and *Sals* (Figure 1) indicated that the shape of their distributions was very similar. All of the phenotypic traits displayed evidence of transgressive segregation [*i.e.* the range of trait values of the RILs exceeded that of the parental lines (Figure 1)].

**Table 1:** Mixed-model ANOVA results partitioning variation among RILs into genetic (V_g_) and phenotypic (V_p_) variance

V_g_		V_p_		H^2^		H^2^_line_		CV_g_		Line Significance	
Trait	*Naut*	*Sals*	*Naut*	*Sals*	*Naut*	*Sals*	*Naut*	*Sals*	*Naut*	*Sals*	*Naut*	*Sals*
Leaf number	8.21	10.52	51.31	55.43	0.16	0.19	0.50	0.55	15.82	17.84	<0.001	<0.001
Days to flowering	14.23	23.52	91.44	118.42	0.16	0.20	0.48	0.55	9.69	12.15	<0.001	<0.001
Fruit number	8.37	12.04	31.63	41.38	0.26	0.29	0.65	0.68	22.36	26.76	<0.001	<0.001
Average fruit weight	0.0003	0.0003	0.001	0.0008	0.33	0.47	0.69	0.75	19.01	19.25	<0.001	<0.001
Shoot weight	0.041	0.10	0.13	0.17	0.31	0.59	0.70	0.88	39.44	58.39	<0.001	<0.001
Root weight	0.014	0.02	0.08	0.06	0.17	0.30	0.52	0.71	22.23	25.78	<0.001	<0.001
Primary branch number	0.081	0.09	0.44	0.43	0.18	0.21	0.54	0.57	20.77	23.11	<0.001	<0.001

Included are broad sense heritability (H^2^ = V_g_/V_p_), heritability on a line-mean basis (H^2^_line_) and the coefficient of genetic variation (CV_g_ = [√V_g_ / trait mean] × 100).

Broad sense heritability was always higher when estimated from RILs grown in the *Sals* treatment (mean H^2^ = 0.32, range = 0.19–0.59) compared with RILs grown in the *Naut* treatment (mean H^2^ = 0.22, range = 0.16–0.33) (Table 1), although both were well within the range of heritabilities for ecologically important traits recently compiled by Hansen *et al.* (2011). Correlations among traits estimated from among-line means are presented in Table S2, Table S3, and Table S4. For the majority of the phenotypic traits measured, the coefficient of genetic variation did not differ substantially between the two rhizobium treatments (Table 1), suggesting that the differences in heritability were due to differences in phenotypic variation or environmental effects. One exception was shoot weight: both heritability and the coefficient of genetic variation were substantially higher in the *Sals* treatment (Table 1). The individual-level variance components and H^2^, estimated by REML, are robust to lack of balance and indicate that significant genetic variance exists to explore with QTL mapping. Heritability on the line-mean basis is also shown in Table 1. As expected, the heritability of the line-mean basis is substantially higher, reflecting the differences in RIL means.

### QTL mapping

**Figure 2:**
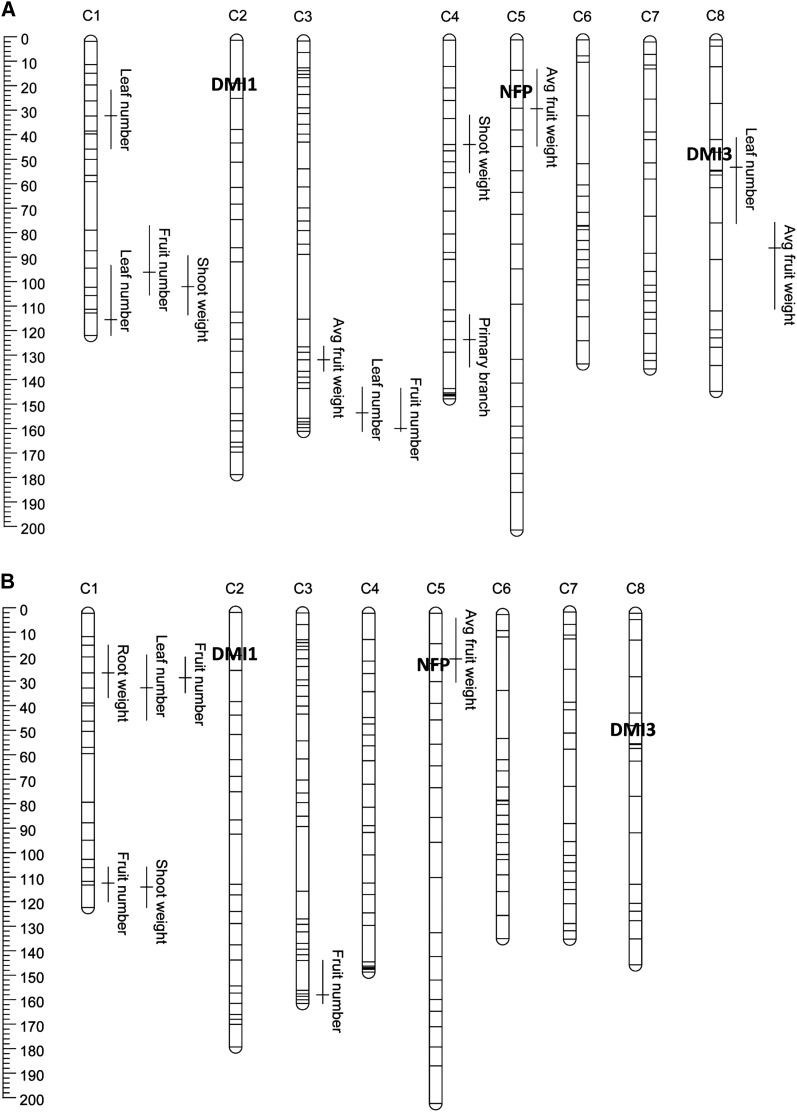
Genomic locations of significant QTL detected for the phenotypic traits of *M. truncatula* when grown with rhizobium strains (A) *Naut* and (B) *Sals*. Chromosome number appears across the top of the linkage groups. The scale on the left indicates the genetic distance between markers in centimorgans (Haldane cM). Each horizontal line represents the position of one genetic marker on the chromosome. Phenotypic trait names appear beside the estimated QTL positions, and the length of the QTL represents the 2-LOD support interval. The vertical line in each QTL indicates where the highest peak was located. The location of the markers representative of the Nod factor signaling genes are in bold.

The final linkage map used for the QTL mapping analyses consisted of 184 markers spanning over eight linkage groups and covering 1215 cM (average of 6.6 cM/marker; see Figure S2 for complete marker orders with genetic distances). All the significant QTL detected in the *Sals* and *Naut* rhizobium treatments are listed in Tables 2 and 3, as well as whether the marker underlying the QTL peak is anchored to the *M. truncatula* genome. In total, we identified eight separate QTL in the *Naut* rhizobium treatment, explaining between 6.9% and 19% of the total genetic variation, depending on the trait examined (Table 2 and Figure 2A). Based on the MCIM results, three of these QTL are potentially pleiotropic: NQTL1-2 had a significant effect on leaf number, number of fruit, and shoot weight; NQTL3-2 had a significant effect on leaf number and number of fruit; and NQTL8 had a significant effect on leaf number and average fruit weight. The direction of NQTL3-2 was opposite for number of fruit and leaf number: the F83005.5 allele increased the former but decreased the latter (Table 2). All the other QTL that affected multiple traits in *Naut* had effects in the same direction. No QTL were identified for days to flowering or root weight in this treatment.

**Table 2:** QTL identified for plant traits collected on *M. truncatula* grown with the *Naut* rhizobium strain

Trait	QTL Name*^a^*	Chr	Position (cM)	Marker	Anchored	LOD	2-LOD Interval (cM)	R^2^	a_0_
Leaf number	NQTL1-1	1	30.4	MTIC448	N	3.1	19.9–43.8	6.9	1.099
	NQTL1-2	1	113.7	MTIC064	Y	4.9	91.5–119.7	10.7	1.400
	NQTL3-2	3	151.8	MTB122	Y	3.4	141.4–159.8	10.7	−1.486
	NQTL8	8	52	DMI3427	Y	3.7	39.9–74.9	7.8	1.217
Number of fruits	NQTL1-2	1	94.3	EM1252.333	N	3.3	75.3–103.6	7.7	1.100
	NQTL3-2	3	158.3	MTIC044	N	4.1	141.8–159.8	7.2	1.123
Average fruit weight	NQTL3-1	3	130.2	MTB6	Y	5	124.6–134.8	14.1	−0.009
	NQTL5	5	28.1	MTIC148	Y	4.3	12–43.4	10.2	−0.007
	NQTL8	8	84.9	MTB333	Y	2.8	74.5–109.9	6.9	0.006
Shoot weight	NQTL1-2	1	100.2	MTIC146	N	2.9	87.5–111.7	6.4	0.067
	NQTL4-1	4	42.6	GO3.350	N	2.9	30.6–54.1	5.6	−0.064
Primary branch number	NQTL4-2	4	122.3	MTIC186	N	4.8	112.1–133.4	19	0.173

The significant QTL for each trait are listed along with the chromosome, position, marker directly below the QTL peak, whether this marker is anchored to the *M. truncatula* genome (Y/N), LOD score, 2-LOD support interval, percentage variance explained by each QTL (R^2^), and additive effect (a_0_, positive values indicate that F83005.5 alleles increase trait means). Both R^2^ and a_0_ are from the MIM analysis, as CIM can overestimate these values. QTL that share the same QTL number indicate those that are putatively pleiotropic based on the MCIM results.

a QTL were named as follows: rhizobium strain prefix (N = *Naut*) and the chromosome number as a suffix, with an additional number depending whether there were multiple QTL per chromosome.

**Table 3:** QTL identified for plant traits collected on *M. truncatula* grown with the *Sals* rhizobium strain

Trait	QTL Name*^a^*	Chr	Position (cM)	Marker	Anchored	LOD	2-LOD Interval (cM)	R^2^	a_0_
Leaf number	SQTL1-1	1	30.4	MTIC448	N	2.9	17-43.6	6.7	1.358
Number of fruits	SQTL1-1	1	26.3	MTB269	N	5.7	17.9-32.4	10.8	1.697
	SQTL1-2	1	110.1	MTIC285	N	5	103.6-117.7	5.4	1.170
	SQTL3	3	155.9	MTIC371	N	3.4	141.8-159.8	5.3	1.321
Average fruit weight	SQTL5	5	12.2	MTIC078	N	4	2-28.1	16.6	−0.01
Shoot weight	SQTL1-3	1	111.7	MTIC064	Y	4.8	103.6-119.7	3.7	0.069
Root weight	SQTL1-1	1	24.3	MTB269	N	3.1	13-34.4	6.4	0.047

The significant QTL for each trait are listed along with the chromosome, position, marker directly below the QTL peak, whether this marker is anchored to the *M. truncatula* genome (Y/N), LOD score, 2-LOD support interval, percentage variance explained by each QTL (R^2^), and additive effect (a_0_, positive values indicate that F83005.5 alleles increase trait means). Both R^2^ snd a_0_ are from the MIM analysis, as CIM can overestimate these values. QTL that share the same QTL number indicate those that are putatively pleiotropic based on the MCIM results.

a QTL were named as follows: rhizobium strain prefix (S = *Sals*) and the chromosome number as a suffix, with an additional number depending whether there were multiple QTL per chromosome.

In the *Sals* rhizobium treatment, five unique QTL were detected, only 1 of which is putatively pleiotropic: SQTL1-1 influenced leaf number, number of fruit, and root weight (Table 3 and Figure 2B). SQTL1-2 and SQTL1-3 had overlapping 2-LOD intervals, but they were not found to be a single joint QTL under MCIM and therefore are assumed to be closely linked. These QTL were of small effect, explaining between 3.7% and 16.6% of the total genetic variation. In this treatment, no QTL were detected for days to flowering or primary branch number.

### QTL by rhizobium strain interactions

An initial comparison of the QTL found in the two rhizobium strain treatments indicated that four QTL influenced the same traits in both treatments: NQTL1-1 and SQTL1-1 for leaf number, NQTL3-2 and SQTL3 for number of fruit, NQTL5 and SQTL5 for average fruit weight, and NQTL1-2 and SQTL1-3 for shoot weight (Tables 2 and 3). However, some of these common QTL differed in the number of phenotypic traits they affected; for example, NQTL1-1 only affected leaf number in *Naut*, but the same QTL influenced leaf number, fruit number, and root weight in *Sals*. Furthermore, QTL for basal branch number were found only in *Naut*, and QTL for root weight were found only in *Sals*. Despite these apparent differences in QTL between the two strain treatments, no significant QTL × rhizobium strain interactions were detected for any of the measured phenotypic traits (*P* = 0.23–0.97).

### Epistatic interactions

Using traditional *P*-values and Holland’s (1998) suggested correction for multiple testing, we would have detected between 13 and 50 marker × marker interactions per trait, but the overwhelming majority were deemed to be false discoveries. We detected only one significant marker × marker interaction that survived FDR, and it influenced root weight in *Naut* (*P* = 0.0000036). It had a partial-r^2^ of 13% (Figure 4) and was found on chromosome 6 between markers E14M60.407 and E12M49.265, which are 8.9 cM apart (Figure S2). The percentage of lines in the four allelic classes were as follows (where P1 = parental allele F83005.5 and P2 = parental allele DZA045.4): E14M60.407-P1 × E12M49.265-P1 = 36%, E14M60.407-P1 × E12M49.265-P2 = 6%, E14M60.407-P2 × E12M49.265-P2 = 53%, E14M60.407-P2 × E12M49.265-P1 = 5%, suggesting the Beavis effect may have overestimated the effect size of the interaction (Beavis 1998). Neither marker was found within the support intervals of the detected additive QTL, nor was there evidence of QTL × QTL × E (*P* = 0.38).

### QTL mapping across strain treatments

**Figure 3:**
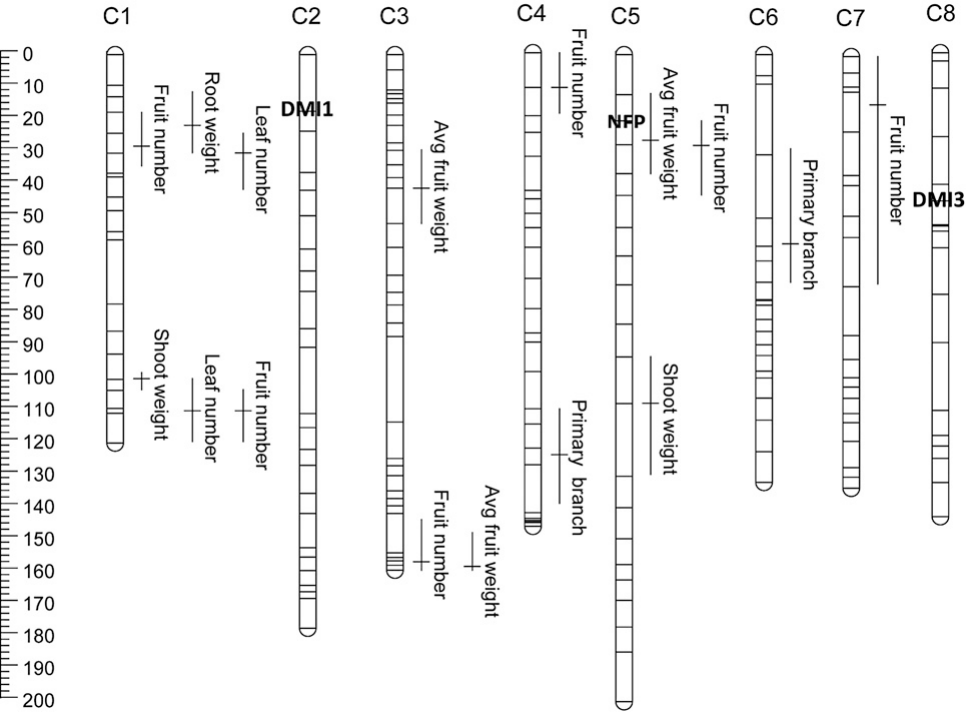
Genomic locations of significant QTL detected for the phenotypic traits of *M. truncatula* averaged across rhizobium strains. Chromosome number appears across the top of the linkage groups. The scale on the left indicates the genetic distance between markers in centimorgans (Haldane cM). Each horizontal line represents the position of one genetic marker on the chromosome. Phenotypic trait names appear beside the estimated QTL positions, and the length of the QTL represents the 2-LOD support interval. The vertical line in each QTL indicates where the highest peak was located. The location of the markers representative of the Nod factor signaling genes are in bold.

In the QTL analyses performed using data averaged across the strain treatments, we found 11 main-effect QTL, each explaining between 3.2% and 20.3% of the total genetic variation (Table 4 and Figure 3). Four QTL are putatively pleiotropic. Six of the QTL found were previously detected in strain-specific analyses (results described above). In addition, new QTL were found: QTL4-1 and QTL7 significantly affected number of fruit, QTL3-1 affected average fruit weight, and QTL5-2 affected shoot weight. The presence of new QTL in this analysis may be due to more precise estimates of the RIL means due to greater within-RIL sample sizes. No QTL were detected for days to flowering.

**Table 4:** QTL identified for plant traits collected on *M. truncatula* averaged over both rhizobium strains

Trait	QTL Name*^a^*	Chr	Position (cM)	Marker	Anchored	LOD	2-LOD Interval (cM)	R^2^	a_0_
Leaf number	QTL1-1	1	30.4	MTIC448	N	3.9	24.3–41.6	9.2	1.237
	QTL1-2	1	110.1	MTIC285	N	4.2	100.2–119.7	10.5	1.248
Number of fruits	QTL1-1	1	28.3	MTB269	N	4.9	17.9–34.4	11.5	1.344
	QTL1-2	1	110.1	MTIC285	N	4	103.6–119.7	8.6	1.200
	QTL3-2	3	156.9	MTIC237	N	3.4	143.8–159.7	7.5	1.071
	QTL4-1	4	10.7	MTIC033	N	2.8	0–18.7	6.5	−1.042
	QTL5-1	5	28.1	MTIC148	Y	2.8	20.5–43.4	7.1	0.853
	QTL7	7	14.9	MTIC147	N	2.9	0–70.3	3.2	0.931
Average fruit weight	QTL3-1	3	41.3	MTIC124	N	3.4	29.5–52.2	8.4	−0.005
	QTL3-2	3	158.3	MTIC044	N	3.1	147.8–159.7	18	−0.009
	QTL5-1	5	26.5	NFP1660	Y	5.1	12–36.8	14.4	−0.008
Shoot weight	QTL1-2	1	100.2	MTIC146	N	5.6	98.3–103.6	5.9	0.074
	QTL5-2	5	107.8	MTB310	Y	3.6	93.4–129.8	8.1	−0.089
Root weight	QTL1-1	1	21.9	MTB46	Y	3.4	11.5–30.3	7.8	0.046
Primary branch number	QTL4-2	4	124.3	MTIC186	N	6.4	110.1–139.4	20.3	0.162

The significant QTL for each trait are listed along with the chromosome, position, marker directly below the QTL peak, whether this marker is anchored to the *M. truncatula* genome (Y/N), LOD score, 2-LOD support interval, percentage variance explained by each QTL (R^2^), and additive effect (a_0_, positive values indicate that F8305.5 alleles increase trait means). Both R^2^ and a_0_ are from the MIM analysis, as CIM can overestimate these values. QTL that share the same QTL number indicate those that are putatively pleiotropic based on the MCIM results.

a QTL were named as follows: chromosome number as a suffix, with an additional number depending whether there were multiple QTL per chromosome.

### Mapping of symbiotic signaling genes and colocalization analysis

Two of the symbiotic signaling genes, *NFP* and *DMI3*, colocalized with two unique QTL (see Figures 2 and 3 and Figure S2 for positions). NFP1660 was found in the interval of a QTL that influenced average fruit weight on chromosome 5 in all treatments (NQTL-5, SQTL5, QTL5-1, Tables 2–4). The effect sizes of these QTL were small; however, they were consistently detected across all analyses. Similarly, DMI31441 was found in the interval of a QTL affecting leaf number in *Naut* only (NTQL8, Table 2); despite its small additive effect, DMI3427 was the marker directly underlying the QTL peak.

The chances that any QTL would colocalize with *NFP*, *DMI1*, or *DMI3* by chance alone were appreciable (*NFP*: *P* = 0.23 for *Naut*, 0.11 for *Sals*, 0.23 for Across-strain; *DMI1*: *P* = 0.22 for *Naut*, 0.06 for *Sals*, 0.14 for Across-strain; *DMI3*: *P* = 0.26 for *Naut*, 0.17 for *Sals*, 0.37 for Across-strain), likely due to the number of traits mapped, number of QTL detected, and the wide confidence intervals. For the specific traits that actually mapped to the candidate genes, however, a slightly different picture emerges. Given the number of QTL detected for average fruit weight and their associated intervals, the odds that one would overlap *NFP* by chance alone were much smaller (*P* = 0.089 for *Naut*, 0.0172 for *Sals*, 0.0459 for Across-strain). For leaf number in the *Naut* treatment, the odds that a QTL would overlap *DMI3* by chance alone were higher (*P* = 0.15).

## Discussion

Mutualisms are known to be genetically variable, where the fitness of one or both partners is dependent on the genotype of its interacting partner, *i.e.* G × G interactions [*e.g.* Hoeksema and Thompson (2007) and Heath (2010)]. These interactions have been found in the legume-rhizobium mutualism (Mhadhbi *et al.* 2005; Laguerre *et al.* 2007; Heath and Tiffin 2007; Rangin *et al.* 2008; Heath *et al.* 2010; Heath 2010); however, to date little is known about the genomic regions responsible for such genetic variation in fitness or whether the loci influencing fitness are partner specific and dependent on the genotype of the interacting partner.

In this study, we performed QTL mapping in two genotypically distinct rhizobium treatments to map additive or epistatic QTL for plant fitness traits and to determine whether they were differentially detected depending on the rhizobium genotype. Three major results emerged from the experiment: (1) QTL for plant fitness and growth traits colocalized with previously described signaling genes; (2) We detected several QTL that appeared to affect multiple phenotypic traits, suggestive of pleiotropy or tight linkage; and (3) We detected no evidence for G × G interactions at the line × rhizobium strain or at the QTL × rhizobium strain level. We discuss these results in turn.

### Colocalization of symbiotic signaling genes

Legumes require rhizobia to survive and reproduce, and the formation of root nodules is an essential component to the establishment of the symbiosis. Among the genes involved in symbiotic signaling [see Kouchi *et al.* (2010) for a review], *NFP*, *DMI1*, *DMI2*, *DMI3*, and *NIN* are involved in the earliest stages of the Nod factor recognition and signaling pathway in *M. truncatula*, and they are essential to the initiation and subsequent formation of nodules between the plant and rhizobia (Schauser *et al.* 1999; Catoira *et al.* 2000; Amor *et al.* 2003; Jones *et al.* 2007). The colocalization of *NFP* and *DMI3* with average fruit weight and leaf number indicates that naturally occurring variation in nodulation signaling genes may potentially influence plant performance and fitness traits. *NFP* and *DMI3* are both required for the initiation of the nodulation pathway, and lab-induced mutations in either of these genes prevents the formation of nodules altogether (Catoira *et al.* 2000; Amor *et al.* 2003). Species-wide sampling in *M. truncatula* has uncovered patterns of polymorphism at both *NFP* and *DMI3* that are consistent with historical purifying selection (De Mita *et al.* 2007). De Mita *et al.* (2006, 2007) have hypothesized that such purifying selection might have maintained high specificity of recognition and removed mutations deleterious to the establishment of symbiosis. Our results suggest that contemporary genetic variation at Nod factor signaling genes may potentially influence plant fitness in *M. truncatula*. Although much is known about the genes critical for legume-rhizobium signaling establishment (Riely *et al.* 2006; Stacey *et al.* 2006), surprisingly little is known about the fitness effects of natural variation at these genes, particularly in the *M. truncatula-Sinorhizobium* interaction.

The colocalization of *NFP* and, to a lesser extent, *DMI3* with QTL for plant performance traits supports the hypothesis that variation in signaling genes can affect plant growth and fitness. For *NFP*, the small probability that a QTL for average fruit weight would colocalize with it by chance alone and the observation that it was a QTL detected in all three analyses (*Naut*, *Sals*, and Across-strain), suggest it is a promising region and gene for further study. If the causal variant is in fact *NFP*, the QTL suggest that one of the parental lines is better able to attract and incorporate (at least these two) rhizobium partners in root nodules, which in turn translates to higher fitness. The presence of the QTL mapping to *NFP* suggests the hypothesis that variation in Nod factor signaling efficiency can exist between genetically different individuals. Nevertheless, these results should be interpreted with caution without further investigation of the loci underlying the QTL intervals. Colocalization of the QTL with *NFP* does not imply causality: QTL intervals contain many genes, and *NFP* may simply be in tight linkage with the loci influencing the plant fitness traits. Regardless of whether *NFP* is the causal locus, these data suggest that the overall region is a promising area for future investigation of loci influencing fruit weight.

### QTL architecture of phenotypic traits

We detected several QTL in the two rhizobium strain treatments (Tables 2 and 3). All of the phenotypic traits displayed evidence of transgressive segregation (Figure 1), suggesting that these traits are controlled by many genes of small effect. The most likely explanation for the observed transgressive segregation is antagonistic QTL [*i.e.* QTL with effects that are in the opposite direction to the parental differences for that trait (Rieseberg *et al.* 2003)]. Between the two strain treatments, half of the measured phenotypes had at least one antagonistic QTL (*Naut* = leaf number, average fruit weight, shoot weight; *Sals* = number of fruit, shoot weight; Tables 2 and 3). It is probable that some QTL of small effect were not detected due to either low power or low-to-moderate heritability, both of which may explain the missing antagonistic QTL for the remaining traits displaying transgressive segregation.

Pleiotropy or tight linkage can both be potential explanations for QTL influencing multiple phenotypes. In this experiment, we used MCIM to formally test for pleiotropy at the QTL level. We found several putatively pleiotropic QTL, and all of their effects match the patterns evident in the line means. For example, our results suggest that in the *Naut* treatment, NQTL1-2 is a pleiotropic QTL increasing the trait values of leaf number, number of fruit, and shoot number; plant weight was positively correlated with fruit number and fruit number is positively correlated with leaf number (Table S3 for correlations), *i.e.* larger plants make more fruit. This region may indicate the location of a QTL controlling a trait common to those phenotypes (*e.g.* growth rate or plant size or plant fitness). Similarly, some pleiotropic QTL influenced multiple traits but in opposite directions (*e.g.* NQTL3-2 in *Naut*). Such antagonistic QTL can result from true antagonistic pleiotropy [*e.g.* Todesco *et al.* (2010)] or from multiple, tightly linked QTL that have opposite effects on two traits. Fine-mapping often shows that single QTL will break into multiple, closely linked QTL, which in turn frequently act in opposite directions [*e.g.* Steinmetz *et al.* (2002) and Kroymann and Mitchell-Olds (2005)]. It is worth noting even MCIM does not necessarily distinguish between one QTL having pleiotropic effects on both traits and two (or more) closely linked QTL each having an effect on one trait only (Jiang and Zeng 1995); however, it is a better estimate of pleiotropic QTL than CIM alone.

Our across-strain analysis identified two additional QTL with opposite effects: QTL3-2 and QTL5-1 increased number of fruit but decreased average fruit weight (Table 4). These two traits are negatively correlated (*r* = −0.32, *P* < 0.0001, Table S5). The parental lines might represent two different reproductive strategies: making fewer heavier fruit (quality) or many smaller fruit (quantity). The trade-off detected is consistent with well-known life history trade-offs in plants [*e.g.* Harper *et al.* (1970), Werner and Platt (1976), and Jakobsson and Eriksson (2000)].

**Figure 4:**
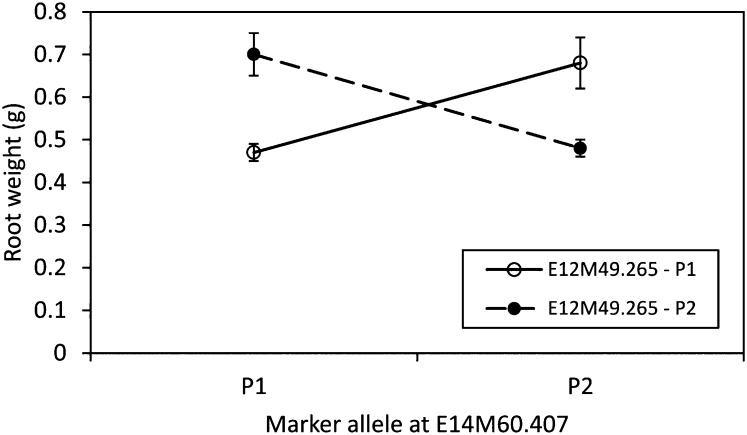
Epistatic QTL detected between markers E12M49.265 and E14M60.407 in Naut, where P1 = F83005.5 and P2 = DZA045.5. The least-square means of each genotype combination are shown with ± SE.

To fully understand the genetic architecture of any trait, it is necessary to understand epistasis, defined as the nonadditive interactions between alleles of different genes [see Phillips (2008) for a review]. Numerous recent studies have tested for the presence of epistatic QTL, *i.e.* marker × marker interactions which have significant effects on phenotypic traits [*e.g.* Leips and Mackay (2000), Weinig *et al.* (2003), Malmberg *et al.* (2005), and Brock *et al.* (2009)]. Here, we tested all pairwise combinations of markers and found only one marker × marker interaction (for root weight in *Naut*, Figure 4). In general, the low frequency of epistatic QTL indicates that epistasis is relatively rare in this mapping population, at least when plants are grown under greenhouse conditions. Nevertheless, the QTL displayed an interesting crossing reaction norm between the two markers: RILs that have alleles from the same parents for both markers have much lower root weight than those that have alleles from alternate parents. Although this epistatic QTL displayed no evidence for QTL × QTL × E, it would be interesting to see if the effect of the interaction changed with other rhizobium genotypes, as root weight is likely to be correlated with rhizobium fitness traits.

When testing for all possible marker × marker interactions in Epistacy, multiple testing becomes a problem. Rather than controlling for the number of linkage groups, as suggested by Holland (1998), a more straightforward way is to use FDR to control for false positives (Benjamini and Hochberg 1995). In future studies, we recommend implementing the latter method for detecting epistatic QTL in Epistacy.

### Mapping G × G interactions

Despite preliminary data that suggested otherwise (Figure S1), we found no evidence for G × G interactions for plant fitness traits in this population; nor did we find any evidence of QTL × rhizobium strain interaction or RIL × rhizobium strain interaction. One implication of this experiment is that it illustrates the difficulties associated with trying to locate the genetic basis of context-dependent traits.

There are a few potential explanations for the lack of G × G interactions detected in this experiment. Two possible hypotheses are (1) the parental lines do not exhibit any form of G × G interactions and thus neither do the RILs; and (2) G × G interactions are due to small-effect loci that were undetected. Another explanation is that only two plant × rhizobium genotype combinations were tested. Previous studies that have found evidence of G × G interactions in the legume-rhizobia symbiosis investigated substantially more unique genotype × genotype combinations compared with the number used in this experiment [*e.g.* Heath and Tiffin (2007), Laguerre *et al.* (2007), Rangin *et al.* (2008), Heath (2010), and Heath *et al.* (2010)]. Although we assayed many RILs, these lines represent the rearrangement of only two parental genomes (Broman 2005). Therefore, from a simplified view, this experiment was a 2 × 2 factorial design, represented by the two parental lines and two rhizobium genotypes. Thus, we might expect to find that only large-effect loci underlying G × G interactions will be found because there are only a few genotype-genotype combinations and there is less statistical power to detect G × G interactions. If population-wide G × G interactions are due to multiple small-effect loci that are distributed throughout the plant and rhizobium genomes, the probability of detecting such G × G interactions in traditional QTL mapping experiments using RILs will be very low.

An alternative approach to isolating the genomic regions influencing G × G interactions would be to perform nested association mapping (NAM) (Yu *et al.* 2008). NAM combines the advantages of using experimental crosses in traditional QTL mapping with the high resolution of association mapping to resolve quantitative traits to their causal loci. Compared with the design used in the present study, NAM would allow significantly more legume × rhizobium genotypes to be tested and provide greater statistical power to detect potential small-effect loci influencing G × G interactions. The time and cost of such a mapping design are considerable; however; the potential outcomes would be invaluable to the study of G × G interactions and thus to understanding the molecular basis of mutualism coevolution.

## Supplementary Material

Supporting Information

## References

[bib1] Amor B. B. Shaw S. L. Oldroyd G. E. D. Maillet F. Penmetsa V. R., 2003 The *NFP* locus of *Medicago truncatula* controls an early step of Nod factor signal transduction upstream of a rapid calcium flux and root hair deformation. Plant J. 34: 495–506 12753588 10.1046/j.1365-313x.2003.01743.x

[bib2] Ané J.-M. Kiss G. B. Riely B. K. Penmetsa V. R. Oldroyd G. E. D., 2004 *Medicago truncatula DMI1* required for bacterial and fungal symbioses in legumes. Science 303: 1364–1367 14963334 10.1126/science.1092986

[bib3] Ané J.-M. Zhu H. Frugoli J., 2008 Recent advances in *Medicago truncatula* genomics. Int. J. Plant Genomics 2008: 256597 18288239 10.1155/2008/256597 PMC2216067

[bib4] Arrighi J.-F. Barre A. Amor B. B. Bersoult A. Soriano L. C., 2006 The *Medicago truncatula* lysin motif-receptor-like kinase gene family includes *NFP* and new nodule-expressed genes. Plant Physiol. 142: 265–279 16844829 10.1104/pp.106.084657 PMC1557615

[bib5] Bailly X. Olivieri I. De Mita S. Cleyet-Marel J.-C. Béna G., 2006 Recombination and selection shape the molecular diversity pattern of nitrogen-fixing *Sinorhizobium* sp. associated to *Medicago*. Mol. Ecol. 15: 2719–2734 16911196 10.1111/j.1365-294X.2006.02969.x

[bib6] Bataillon T. Ronfort J., 2006 Evolutionary and ecological genetics of *Medicago truncatula. Medicago truncatula* Handbook. Available at: http://www.noble.org/Global/medicagohandbook/pdf/EvolutionaryEcologicalGenetics.pdf

[bib7] Beavis W. D., 1998 QTL analyses: Power, precision and accuracy, pp. 145–162 in Molecular Dissection of Complex Traits, edited by Patterson A. H. CRC Press, New York

[bib8] Benjamini Y. Hochberg Y., 1995 Controlling the false discovery rate: a practical and powerful approach to multiple testing. J. R. Stat. Soc., B 57: 289–300

[bib9] Beringer J. E., 1974 R factor transfer in *Rhizobium leguminosarum*. J. Gen. Microbiol. 84: 188–198 4612098 10.1099/00221287-84-1-188

[bib10] Bever J. D., 1999 Dynamics within mutualism and the maintenance of diversity: inference from a model of interguild frequency dependence. Ecol. Lett. 2: 52–61

[bib11] Bonnin I. Prosperi J.-M. Olivieri I., 1996 Genetic markers and quantitative genetic variation in *Medicago truncatula* (Leguminosae): a comparative analysis of population structure. Genetics 143: 1795–1805 8844165 10.1093/genetics/143.4.1795 PMC1207440

[bib12] Boucher D. B. (Editor), 1985 The Biology of Mutualism: Ecology and Evolution. Oxford University Press, New York

[bib13] Branca A. Paape T. D. Zhou P. Briskine R. Farmer A. D., 2011 Whole-genome nucleotide diversity, recombination, and linkage disequilibrium in the model legume *Medicago truncatula*. Proc. Natl. Acad. Sci. USA 108: E864–E870 21949378 10.1073/pnas.1104032108 PMC3198318

[bib14] Brock M. T. Stinchcombe J. R. Weinig C., 2009 Indirect effects of FRIGIDA: floral trait (co)variances are altered by seasonally variable abiotic factors associated with flowering time. J. Evol. Biol. 22: 1826–1838 19583697 10.1111/j.1420-9101.2009.01794.x

[bib15] Broman K. W., 2005 The genomes of recombinant inbred lines. Genetics 169: 1133–1146 15545647 10.1534/genetics.104.035212 PMC1449115

[bib16] Bronstein J. L., 1994 Our current understanding of mutualism. Q. Rev. Biol. 69: 31–51

[bib17] Bronstein J. L., 2001 The costs of mutualism. Am. Zool. 41: 127–141

[bib18] Catoira R. Galera C. de Bailly F. Penmetsa R. V. Journet E. P., 2000 Four genes of *Medicago truncatula* controlling components of a nod factor transduction pathway. Plant Cell 12: 1647–1666 11006338 10.1105/tpc.12.9.1647 PMC149076

[bib19] Churchill G. A. Doerge R. W., 1994 Empirical threshold values for quantitative trait mapping. Genetics 138: 963–971 7851788 10.1093/genetics/138.3.963 PMC1206241

[bib20] Cook D. R., 1999 *Medicago truncatula*—a model in the making! Commentary Curr. Opin. Plant Biol. 2: 301–304 10459004 10.1016/s1369-5266(99)80053-3

[bib21] De Mita S. Santoni S. Hochu I. Ronfort J. Bataillon T., 2006 Molecular evolution and positive selection of the symbiotic gene *NORK* in *Medicago truncatula*. J. Mol. Evol. 62: 234–244 16474986 10.1007/s00239-004-0367-2

[bib22] De Mita S. Ronfort J. McKhann H. I. Poncet C. El Malki R., 2007 Investigation of the demographic and selective forces shaping the nucleotide diversity of genes involved in nod factor signaling in *Medicago truncatula*. Genetics 177: 1–3 18073426 10.1534/genetics.107.076943 PMC2219494

[bib23] Emrich K. Wilde F. Miedaner T. Piepho H. P., 2008 REML approach for adjusting *Fusarium* head blight rating to a phenological data in inoculated selection experiments of what. Theor. Appl. Genet. 117: 65–73 18392606 10.1007/s00122-008-0753-z

[bib24] Endré G. Kereszt A. Kevei Z. Mihacea S. Kalo P., 2002 A receptor kinase gene regulating symbiotic nodule development. Nature 417: 962–966 12087406 10.1038/nature00842

[bib25] Gomulkiewicz R. Drown D. M. Dybdahl M. F. Godsoe W. Nuismer S. L., 2007 Dos and don’ts of testing the geographic mosaic theory of coevolution. Heredity 98: 249–258 17344805 10.1038/sj.hdy.6800949

[bib26] Hadfield J. D. Wilson A. J. Garant D. Sheldon B. C. Kruuk L. E., 2010 The misuse of BLUP in ecology and evolution. Am. Nat. 175: 116–125 19922262 10.1086/648604

[bib27] Hall T. M., 1999 BioEdit: a user-friendly biological sequence alignment editos and analysis program for Windows 95/98/NT. Nucleic Acids Symposium Series 41: 95–98

[bib28] Hamon C. Baranger A. Miteul H. Lecointe R. Le Goff I., 2010 A complex genetic network involving a broad-spectrum locus and strain-specific loci controls resistance to different pathotypes of *Aphanomyces euteiches* in *Medicago truncatula*. Theor. Appl. Genet. 120: 955–970 20012740 10.1007/s00122-009-1224-x

[bib29] Hansen T. F. Pélabon C. Houle D., 2011 Heritability is not evolvability. Evol. Biol. 38: 258–277

[bib30] Harper A. J. L. Lovell P. H. Moore K. G., 1970 The shapes and sizes of seeds. Annu. Rev. Ecol. Syst. 1: 327–356

[bib31] Heath K. D. Tiffin P., 2007 Context dependence in the coevolution of plant and rhizobial mutualists. Proc. Biol. Sci. 274: 1905–1912 17535796 10.1098/rspb.2007.0495 PMC2270936

[bib32] Heath K. D., 2010 Intergenomic epistasis and coevolutionary constraint in plants and rhizobia. Evolution 64: 1446–1458 20002161 10.1111/j.1558-5646.2009.00913.x

[bib33] Heath K. D. Stock A. J. Stinchcombe J. R., 2010 Mutualism variation in the nodulation response to nitrate. J. Evol. Biol. 23: 1–7 20825525 10.1111/j.1420-9101.2010.02092.x

[bib34] Hoeksema J. D. Thompson J. N., 2007 Geographic structure in a widespread plant-mycorrhizal interaction: pines and false truffles. J. Evol. Biol. 20: 1148–1163 17465924 10.1111/j.1420-9101.2006.01287.x

[bib35] Holland J. B., 1998 EPISTACY: a SAS program for detecting two-locus epistatic interactions using genetic marker information. J. Hered. 89: 374–375

[bib36] Holland J. B. Nyquist W. E. Cervantes-Martinez G. T., 2003 Estimating and interpreting heritability for plant breeding: an update, pp. 9–112 in *Plant Breeding Reviews*, Vol. 22, edited by J. Janick. John Wiley and Sons, Oxford, UK.

[bib37] Jakobsson A. Eriksson O., 2000 A comparative study of seed number, seed size, seedling size and recruitment in grassland plants. Oikos 88: 494–502

[bib38] Jansen R. C., 1993 Interval mapping of multiple quantitative trait loci. Genetics 135: 205–211 8224820 10.1093/genetics/135.1.205 PMC1205619

[bib39] Jiang J. Zeng Z.-B., 1995 Multiple trait analysis of genetics mapping for quantative trait loci. Genetics 140: 1111–1127 7672582 10.1093/genetics/140.3.1111 PMC1206666

[bib40] Jones K. M. Kobayashi H. Davies B. W. Taga M. E. Walker G. C., 2007 How rhizobial symbionts invade plants: the *Sinorhizobium*-*Medicago* model. Nat. Rev. Microbiol. 5: 619–633 17632573 10.1038/nrmicro1705 PMC2766523

[bib41] Kao C.-H. Zend Z.-B. Teasdale R. D., 1999 Multiple interval mapping for quantitative trait loci. Genetics 152: 1203–1216 10388834 10.1093/genetics/152.3.1203 PMC1460657

[bib42] Keurentjes J. J. B. Bentsink L. Alonso-Blanco C. Hanhart C. J. Blankestijn-De Vries H., 2007 Development of a near-isogenic line population of *Arabidopsis thaliana* and comparison of mapping power with a recombinant inbred line population. Genetics 175: 891–905 17179089 10.1534/genetics.106.066423 PMC1800614

[bib43] Kouchi H. Imaizumi-Anraku H. Hayashi M. Hakoyama T. Nakagawa T., 2010 How many peas in a pod? Legume genes responsible for mutualistic symbioses underground. Plant Cell Physiol. 51: 1381–1397 20660226 10.1093/pcp/pcq107 PMC2938637

[bib44] Kroymann J. Mitchell-Olds T., 2005 Epistasis and balanced polymorphism influencing complex trait variation. Nature 435: 95–98 15875023 10.1038/nature03480

[bib45] Laguerre G. Depret G. Bourion V. Duc G., 2007 *Rhizobium leguminosarum* bv. *viciae* genotypes interact with pea plants in developmental responses of nodules, roots and shoots. New Phytol. 176: 680–690 17822397 10.1111/j.1469-8137.2007.02212.x

[bib46] Leips J. Mackay T. F., 2000 Quantitative trait loci for life span in *Drosophila melanogaster*: interactions with genetic background and larval density. Genetics 155: 1773–1788 10924473 10.1093/genetics/155.4.1773 PMC1461186

[bib47] Lévy J. Bres C. Geurts R. Chalhoub B. Kulikova O., 2004 A putative Ca2+ and calmodulin-dependent protein kinase required for bacterial and fungal symbioses. Science 303: 1361–1364 14963335 10.1126/science.1093038

[bib48] Long J. Holland J. B. Munkvold G. P. Jannink J.-L., 2006 Responses to selection for partial resistance to crown rust in oat. Crop Sci. 46: 1260–1265

[bib49] Loridon K. PcPhee K. Morin J. Dubreuil P. Pilet-Nayel M.-L., 2005 Microsatellite marker polymorphism and mapping in pea (*Pisum sativum* L.). Theor. Appl. Genet. 111: 1022–1031 16133320 10.1007/s00122-005-0014-3

[bib84] Lynch M. Walsh B., 1998 Genetics and Analysis of Quantitative Traits. Sinauer Associaties, Inc. Sunderland, MA

[bib50] Mackay T. F. C., 2001 Quantitative trait loci in *Drosophila*. Nat. Rev. Genet. 2: 11–20 11253063 10.1038/35047544

[bib51] Malmberg R. L. Held S. Waits A. Mauricio R., 2005 Epistasis for fitness-related quantitative traits in *Arabidopsis thaliana* grown in the field and in the greenhouse. Genetics 171: 2013–2027 16157670 10.1534/genetics.105.046078 PMC1456117

[bib52] Mhadhbi H. Jebara M. Limam F. Huguet T. Aouani M. E., 2005 Interaction between *Medicago truncatula* lines and *Sinorhizobium meliloti* strains for symbiotic efficiency and nodule antioxidant activities. Physiol. Plant. 124: 4–11

[bib53] Moran N., 1981 Intraspecific variability in herbivore performance and host quality: a field study of *Uroleucon caligatum* (Homoptera: Aphididae) and its *Solidago* hosts (Asteraceae). Ecol. Entomol. 6: 301–306

[bib54] Moreau D Salon C Munier-Jolain N., 2006 Using a standard framework for the phenotypic analysis of *Medicago truncatula*: an effective method for characterizing the plant material used for functional genomics approaches. Plant Cell Environ. 29: 1087–1098 17080935 10.1111/j.1365-3040.2005.01483.x

[bib55] Mun J.-H. Kim D.-J. Choi H.-K. Gish J. Debellé F., 2006 Distribution of microsatellites in the genome of *Medicago truncatula*: a resource of genetic markers that integrate genetic and physical maps. Genetics 172: 2541–2555 16489220 10.1534/genetics.105.054791 PMC1456377

[bib56] Parker M. P., 1995 Plant fitness variation caused by different mutualist genotypes. Ecology 76: 1525–1535

[bib57] Phillips P. C., 2008 Epistasis—the essential role of gene interactions in the structure and evolution of genetic systems. Nat. Rev. Genet. 9: 855–867 18852697 10.1038/nrg2452 PMC2689140

[bib58] Piepho H.-P. Möhring J., 2007 Computing heritability and selection response from unbalanced plant breeding trials. Genetics 177: 1881–1888 18039886 10.1534/genetics.107.074229 PMC2147938

[bib59] Rangin C. Brunel B. Cleyet-Marel J.-C. Perrineau M.-M. Béna G., 2008 Effects of *Medicago truncatula* genetic diversity, rhizobial competition, and strain effectiveness on the diversity of a natural sinorhizobium species community. Appl. Environ. Microbiol. 74: 5653–5661 18658290 10.1128/AEM.01107-08 PMC2547051

[bib60] Riely B. K. Mun J. H. Ané J., 2006 Unravelling the molecular basis for symbiotic signal transduction in legumes. Mol. Plant Pathol. 7: 197–207 20507440 10.1111/j.1364-3703.2006.00328.x

[bib61] Rieseberg L. H. Widmer A. Arntz A. M. Burke J. M., 2003 The genetic architecture necessary for transgressive segregation is common in both natural and domesticated populations. Philos. Trans. R. Soc. Lond. B Biol. Sci. 358: 1141–1147 12831480 10.1098/rstb.2003.1283 PMC1693210

[bib62] Salvaudon L. Héraudet V. Shykoff J. A., 2005 Parasite-host fitness trade-offs change with parasite identity: genotype-specific interactions in a plant-pathogen system. Evolution 59: 2518–2524 16526500

[bib63] Schauser L. Roussis A. Stiller J. Stougaard J., 1999 A plant regulator controlling development of symbiotic root nodules. Nature 402: 191–195 10647012 10.1038/46058

[bib64] Service P., 1984 Genotypic interactions in an aphid-host plant relationship: *Uroleucon rudbeckiae* and *Rudbeckia laciniata*. Oecologia 61: 271–276 10.1007/BF00396772 28309423

[bib65] Smith D. C. Douglas A. E. (Editors), 1987 The Biology of Symbiosis. Edward Arnold, London

[bib66] Somasegaran P. Hoben H. J., 1994 Handbook for rhizobia: Methods in Legume-rhizobium Technology, Springer-Verlag, New York

[bib67] Stacey G. Libault M. Brechenmacher L. Wan J. May G. D., 2006 Genetics and functional genomics of legume nodulation. Curr. Opin. Plant Biol. 9: 110–121 16458572 10.1016/j.pbi.2006.01.005

[bib68] Steinmetz L. M. Sinha H. Richards D. R. Spiegelman J. I. Oefner P. J., 2002 Dissecting the architecture of a quantitative trait locus in yeast. Nature 416: 326–330 11907579 10.1038/416326a

[bib70] Storey J. D. Tibshirani R., 2003 Statistical significance for genomewide studies. Proc. Natl. Acad. Sci. USA 100: 9440–9445 12883005 10.1073/pnas.1530509100 PMC170937

[bib71] Todesco M. Balasubramanian S. Hu T. T. Traw M. B. Horton M., 2010 Natural allelic variation underlying a major fitness trade-off in Arabidopsis thaliana. Nature 465: 632–636 20520716 10.1038/nature09083 PMC3055268

[bib72] Van Ooijen J., 1992 Accuracy of mapping quantitative trait loci in autogamous species. Theor. Appl. Genet. 84: 803–811 24201478 10.1007/BF00227388

[bib73] Van Ooijen J., 2006 *JoinMap 4: Software for the Calculation of Genetic Linkage Maps in Experimental Populations* Kyazma B.V., Wageningen, Netherlands

[bib74] Voorrips R. E., 2002 MapChart: software for the graphical presentation of linkage maps and QTLs. J. Hered. 93: 77–78 12011185 10.1093/jhered/93.1.77

[bib75] Wade M. J., 2007 The co-evolutionary genetics of ecological communities. Nat. Rev. Genet. 8: 185–195 17279094 10.1038/nrg2031

[bib76] Wang S. Basten C. J. Zeng Z.-B., 2011 Windows QTL Cartographer V2.5. Available at: http://statgen.ncsu.edu/qtlcart/WQTLCart.htm

[bib77] Weinig C. Stinchcombe J. R. Schmitt J., 2003 QTL architecture of resistance and tolerance traits in *Arabidopsis thaliana* in natural environments. Mol. Ecol. 12: 1153–1163 12694279 10.1046/j.1365-294x.2003.01787.x

[bib78] Werner P. A. Platt W. J., 1976 Ecological relationships of co-occurring goldenrods (Solidago: Compositae). Am. Nat. 110: 959–971

[bib79] Young N. D. Cannon S. B. Sato S. Kim D. Cook D. R., 2005 Sequencing the genespaces of *Medicago truncatula* and *Lotus japonicus*. Plant Physiol. 137: 1174–1181 15824279 10.1104/pp.104.057034 PMC1088310

[bib80] Young N. D. Debellé F. Oldroyd G. E. D. Geurts R. Cannon S. B., 2011 The Medicago genome provdies insight into the evolution of rhizobial symbioses. Nature 480: 520–524 22089132 10.1038/nature10625 PMC3272368

[bib81] Yu J. Holland J. B. McMullen M. D. Buckler E. S., 2008 Genetic design and statistical power of nested association mapping in maize. Genetics 178: 539–551 18202393 10.1534/genetics.107.074245 PMC2206100

[bib82] Zeng Z.-B., 1993 Theoretical basis for separation of multiple linked gene effects in mapping quantitative trait loci. Proc. Natl. Acad. Sci. USA 90: 10972–10976 8248199 10.1073/pnas.90.23.10972 PMC47903

[bib83] Zeng Z.-B., 1994 Precision mapping of quantitative trait loci. Genetics 136: 1457–1468 8013918 10.1093/genetics/136.4.1457 PMC1205924

