# Supplementary material for: Mapping the Genetic Basis of Symbiotic Variation in Legume-Rhizobium Interactions in Medicago truncatula
Source: G3 (Bethesda). 2012 Nov 1;2(11):1291–303. doi: 10.1534/g3.112.003269 (PMC3484660; doi:10.1534/g3.112.003269)
Supplement: Supporting Information [file supp_2_11_1291__index.html]

Supporting Information 

# Mapping the Genetic Basis of Symbiotic Variation in Legume-Rhizobium Interactions in *Medicago truncatula*

## Supporting Information for Gorton *et al.*, 2012

**Files in this Data Supplement:**

- Supporting Information - Figures S1 and S2, Files S1 and S2, and Tables S1-S7 (PDF, 896 KB)
- Figure S1 - Preliminary leaf count data collected on the parental lines of the LR03 RIL mapping population grown with rhizobium strains *Naut* and *Sals* (PDF, 107 KB)
- Figure S2 - Linkage map of the LR03 RIL mapping population, constructed in JoinMap 4.0 (PDF, 680 KB)
- Table S1 - Primer sequences used for sequencing Nod factor signaling genes in parental lines and RILs (PDF, 61 KB)
- Table S2 - Information on type and location of SNPs used to genotype the LR03 RILs (PDF, 63 KB)
- Table S3 - Correlations between RIL least-square means for all traits in *Naut* environment (PDF, 72 KB)
- Table S4 - Correlations between RIL least-square means for all traits in *Sals* environment (PDF, 72 KB)
- Table S5 - Correlations between RIL least-square means for all traits in the across rhizobium strains analysis (PDF, 64 KB)
- Table S6 - AFLP primer combinations used to generate fingerprints (PDF, 70 KB)
- Table S7 - List of additional Medicago truncatula EST microsatellite primers used in this study (PDF, 70 KB)
- File S2 - Supporting Materials and methods (PDF, 90 KB)
- File S1 - Phenotypic data and marker genotypes (.xlsx, 514 KB)
