# Supplementary material for: Mapping the Genetic Basis of Symbiotic Variation in Legume-Rhizobium Interactions in Medicago truncatula
Source: G3 (Bethesda). 2012 Nov 1;2(11):1291–303. doi: 10.1534/g3.112.003269 (PMC3484660; doi:10.1534/g3.112.003269)
Supplement: Supporting Information [file supp_2.11.1291_FigureS1.pdf]

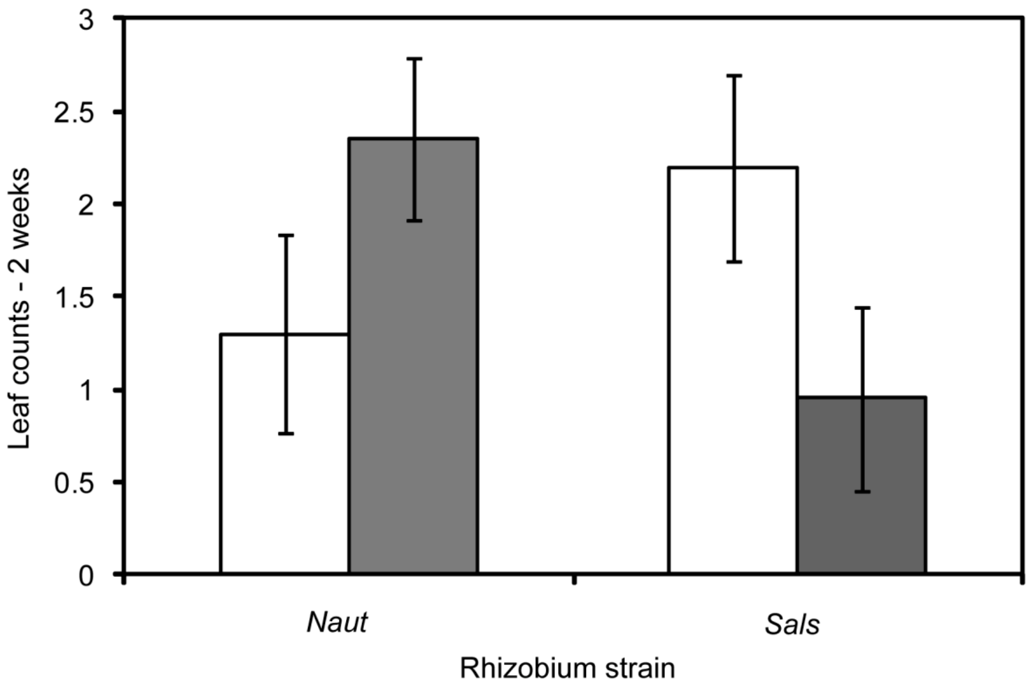

Figure S1 Preliminary leaf count data collected on the parental lines of the LR03 RIL mapping population grown with rhizobium strains *Naut* and *Sals* (Heath, unpublished). White = female parent, line F83005.5; black = male parent, line DZA045.5. Means are shown ± SE (n= 20 for each plant line). Rhizobium strain × parental line = 0.02.
