# Supplementary material for: Mapping the Genetic Basis of Symbiotic Variation in Legume-Rhizobium Interactions in Medicago truncatula
Source: G3 (Bethesda). 2012 Nov 1;2(11):1291–303. doi: 10.1534/g3.112.003269 (PMC3484660; doi:10.1534/g3.112.003269)
Supplement: Supporting Information [file supp_2.11.1291_FigureS2.pdf]

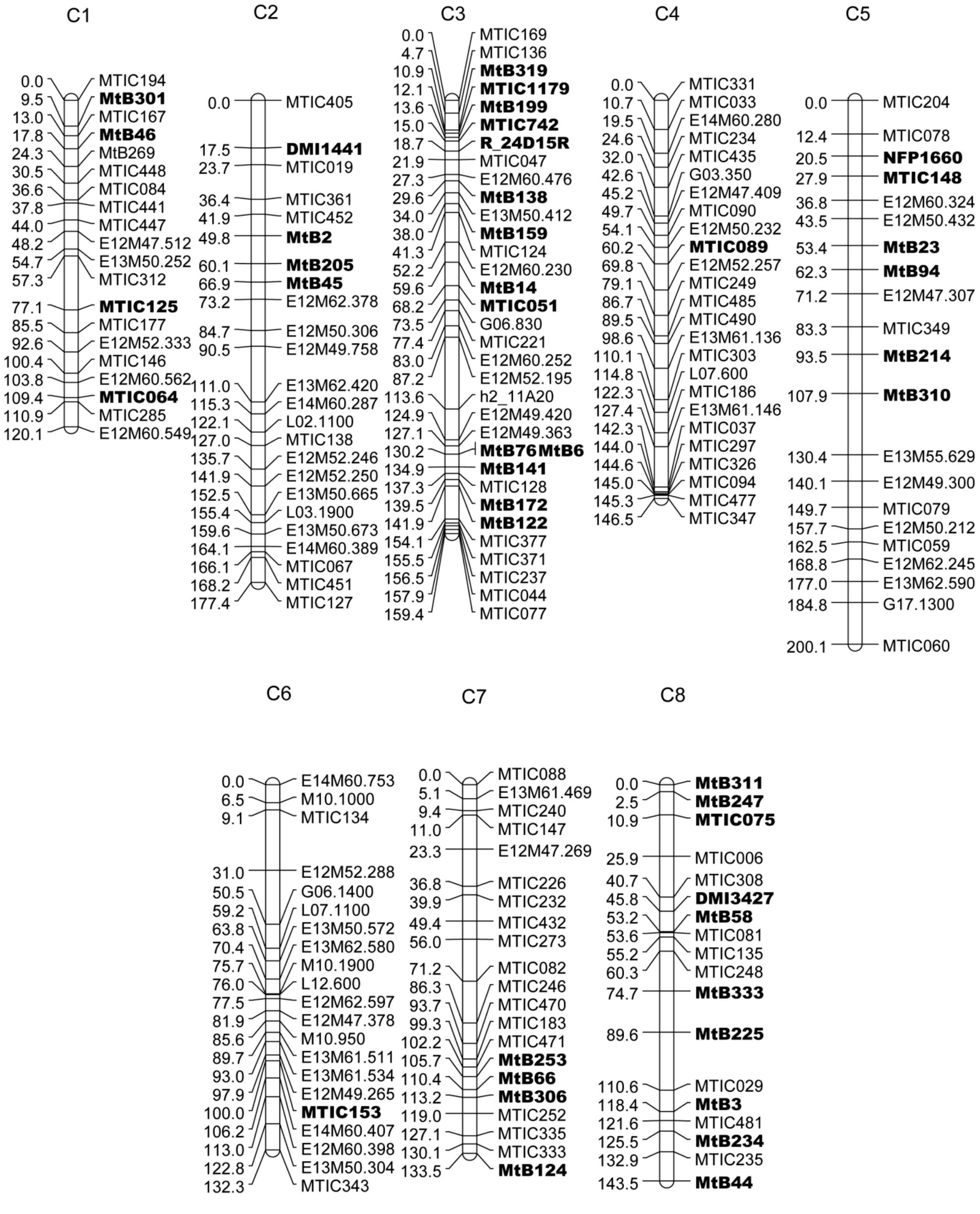

Figure S2 Linkage map of the LR03 RIL mapping population, constructed in JoinMap 4.0. Number of markers =184. Bolded markers are anchored to the *M. truncatula* genome. The genetic distances (in Haldane cM) between markers are indicated on the left side of the linkage groups.
