# Supplementary material for: Mapping the Genetic Basis of Symbiotic Variation in Legume-Rhizobium Interactions in Medicago truncatula
Source: G3 (Bethesda). 2012 Nov 1;2(11):1291–303. doi: 10.1534/g3.112.003269 (PMC3484660; doi:10.1534/g3.112.003269)
Supplement: Supporting Information [file supp_2.11.1291_TableS1.pdf]

Table S1 Primer sequences used for sequencing Nod factor signaling genes in parental lines and RILs . Only those primers indicated with as asterisk were sequenced in the RILs (see Table S2 for details). Fragment length and melting temperature of the primers (T_m; forward, reverse) also included.

Fragments	Forward primer	Reverse primer	Fragment length	T_m (°C)	Source
DMI1					
DMI1-MT1*	AATACATACACATAAAAGGAATC	CATCTACCATATAAGCAACTCT	982 bp	46.5, 49.4	De Mita *et al.* 2007
DMI3					
DMI3.3*	TCTTGAGCTTTGTTCCGGTGGTGA	AGATGTGAGCTACCGTGTTCCCAA	625 bp	60.6, 60.1	This study
DMI3.4	CGTGATGGAACAGTTGACATGCGT	TGTGTGCATTACCCTGAGCATGGA	820 bp	59.9, 60.2	This study
NFP					
NFP-1*	TTACATGCCCTGTGGATTCTCCTC	ATCTGCAGTCTCGGATGAAGT	755 bp	58.6, 59.8	This study
NFP-2	AAGTACTTCATCGTCCGAGACTGC	CTGCCAAAGAAGCCAAACTTAGAGC	785 bp	58.4, 58.5	This study
NIN					
NIN-1	TTGAGGAGCTGTTGGGAGAAGGTT	TCCATTATCTCGTTCACCGCTGCT	760 bp	60.2, 60.3	This study
NIN-2	GTGGTGCATCAGGTTGTGGTGTTT	TACTGCTCTGATCATGCTGCTGCT	827 bp	60.3, 60.1	This study
NIN-4	GTGAAGGCAACTTTCGCGGATGAA	CTGCTGTTGCGGAAAGTGTTTGGA	281 bp	60.1, 60.3	This study
DMI2					
DMI2-1	GGTTGATATTGTCCGCGAGT	TGACAAGGTTTGGGTTGTGA	177 bp	55, 54	This study
DMI2-2	ATATTGTCCGCGAGTTGGAG	ATCTCGGTTGAGGGTGTGAC	188 bp	54.8, 56.8	This study
DMI2-3	CCCCTTTTGAATGCCTATGA	TTTTCTTGGTTGTGCAGCAC	99 bp	52.6, 54.2	This study
DMI2-4	AACTCAGGGAACCCGAGAAT	GTAACCCAGAAG AGGCACCA	65 bp	55.8, 56.8	This study
