# Supplementary material for: Mapping the Genetic Basis of Symbiotic Variation in Legume-Rhizobium Interactions in Medicago truncatula
Source: G3 (Bethesda). 2012 Nov 1;2(11):1291–303. doi: 10.1534/g3.112.003269 (PMC3484660; doi:10.1534/g3.112.003269)
Supplement: Supporting Information [file supp_2.11.1291_TableS2.pdf]

Table S2 Information on type and location of SNPs used to genotype the LR03 RILs

Gene	Polymorphism in parents (Y/N)	Nature of SNP	Position in gene
DMI1	Y	Nonsynonymous, T/C	388 bp
DMI3	Y	Non-coding, A/G	6700 bp
DMI2	N	-	-
NFP	Y	Synonymous, A/G	792 bp
NIN	N	-	-
