# Supplementary material for: Mapping the Genetic Basis of Symbiotic Variation in Legume-Rhizobium Interactions in Medicago truncatula
Source: G3 (Bethesda). 2012 Nov 1;2(11):1291–303. doi: 10.1534/g3.112.003269 (PMC3484660; doi:10.1534/g3.112.003269)
Supplement: Supporting Information [file supp_2.11.1291_TableS3.pdf]

Table S3 Correlations between RIL least-square means for all traits in *Naut* environment. Data presented are the pearson correlation coefficients between least-square line means calculated using PROC CORR in SAS (v.9.2)

	Leaf number	Days to flowering	Fruit number	Average fruit weight	Shoot weight	Root weight	Primary branch number
Leaf number		-0.26 **	0.36****	0.29****	0.07	0.14	0.29****
Days to flowering			0.08	-0.02	0.43****	0.31****	0.34****
Fruit number				-0.31****	0.52****	0.40****	0.21 **
Average fruit weight					-0.11	-0.04	0.10
Shoot weight						0.66****	0.35****
Root weight							0.38****
Primary branch number							

P< 0.0001 = ****, P< 0.001 = ***, P<0.01 = **, P<0.05 = *
