# Supplementary material for: Mapping the Genetic Basis of Symbiotic Variation in Legume-Rhizobium Interactions in Medicago truncatula
Source: G3 (Bethesda). 2012 Nov 1;2(11):1291–303. doi: 10.1534/g3.112.003269 (PMC3484660; doi:10.1534/g3.112.003269)
Supplement: Supporting Information [file supp_2.11.1291_TableS4.pdf]

Table S4 Correlations between RIL least-square means for all traits in *Sals* environment. Data presented are the pearson correlation coefficients between least-square line means calculated using PROC CORR in SAS (v.9.2).

	Leaf number	Days to flowering	Fruit number	Average fruit weight	Shoot weight	Root weight	Primary branch number
Leaf number		-0.36****	0.35****	0.27 **	-0.08	0.11	0.33****
Days to flowering			0.14	-0.08	0.59****	0.50****	0.24 **
Fruit number				-0.19**	0.49****	0.51****	0.23**
Average fruit weight					-0.15*	0.05	0.09
Shoot weight						0.70****	0.18 **
Root weight							0.30****
Primary branch number							

P< 0.0001 = ****, P< 0.001 = ***, P<0.01 = **, P<0.05 = *
