# Supplementary material for: Mapping the Genetic Basis of Symbiotic Variation in Legume-Rhizobium Interactions in Medicago truncatula
Source: G3 (Bethesda). 2012 Nov 1;2(11):1291–303. doi: 10.1534/g3.112.003269 (PMC3484660; doi:10.1534/g3.112.003269)
Supplement: Supporting Information [file supp_2.11.1291_TableS5.pdf]

Table S5 Correlations between RIL least-square means for all traits in the across rhizobium strains analysis. Data presented are the pearson correlation coefficients between least-square line means calculated using PROC CORR in SAS (v.9.2).

	Leaf number	Days to flowering	Fruit number	Average fruit weight	Shoot weight	Root weight	Primary branch number
Leaf number (6 weeks)		-0.29****	0.37****	0.29****	-0.008	0.18 **	0.33****
Days to flowering			0.16 *	-0.04	0.57****	0.46****	0.39****
Fruit number				-0.32****	0.49****	0.51****	0.27 ***
Average fruit weight					-0.13	0.02	0.09
Shoot weight						0.70****	0.31****
Root weight							0.42****
Primary branch number							

P< 0.0001 = ****, P< 0.001 = ***, P<0.01 = **, P<0.05 = *.
