# Supplementary material for: Mapping the Genetic Basis of Symbiotic Variation in Legume-Rhizobium Interactions in Medicago truncatula
Source: G3 (Bethesda). 2012 Nov 1;2(11):1291–303. doi: 10.1534/g3.112.003269 (PMC3484660; doi:10.1534/g3.112.003269)
Supplement: Supporting Information [file supp_2.11.1291_TableS6.pdf]

Table S6 AFLP primer combinations used to generate fingerprints

	E-AC [a]	E-AG	E-AT	E-AGA
M-CAA [b]	E12M47	E13M47 (PB)	E14M47	E39M47
M-CAC	E12M48 (PI [c])	E13M48 (PA)	E14M48 (PD)	E39M48
M-CAG	E12M49	E13M49	E14M49 (PE)	E39M49
M-CAT	E12M50 (PK)	E13M50 (PC)	E14M50	E39M50
M-CCA	E12M51	E13M51	E14M51	E39M51 (PG)
M-CCC	E12M52	E13M52	E14M52	E39M52
M-CCG	E12M53	E13M53	E14M53	E39M53
M-CCT	E12M54	E13M54	E14M54	E39M54
M-CGA	E12M55	E13M55	E14M55	E39M55
M-CGC	E12M56	E13M56	E14M56	E39M56
M-CGG	E12M57	E13M57 (PM)	E14M57	E39M57
M-CGT	E12M58	E13M58	E14M58	E39M58 (PF)
M-CTA	E12M59	E13M59 (PL)	E14M59	E39M59
M-CTC	E12M60	E13M60	E14M60	E39M60
M-CTG	E12M61 (PH)	E13M61	E14M61	E39M61
M-CTT	E12M62 (PJ)	E13M62	E14M62	E39M62

[a] E indicates *Eco*RI primer 5'-GAC TGC GTA CCA ATT C+ANN-3'
[b] M indicates *Mse*I primer 5'-GAT GAG TCC TGA GTA A+CNN-3'
[c] In brackets, code names of *Eco*RI/*Mse*I primer combinations used in THOQUET *et al*. (2002)
