# Supplementary material for: Mapping the Genetic Basis of Symbiotic Variation in Legume-Rhizobium Interactions in Medicago truncatula
Source: G3 (Bethesda). 2012 Nov 1;2(11):1291–303. doi: 10.1534/g3.112.003269 (PMC3484660; doi:10.1534/g3.112.003269)
Supplement: Supporting Information [file supp_2.11.1291_TableS7.pdf]

Table S7 List of additional Medicago truncatula EST microsatellite primers used in this study (Huguet, unpublished). Primer sequences, linkage group in M. truncatula, GenBank EST identification and repeititve motif included.

	Primers (5'-3')		Linkage	GenBank EST	Repetitive
	Forward	Reverse	group	Idenitification	motif
MTIC6	CACAGTACTGCCACCGTCAA	GTCGGAGGTGAGAGGTTGAA	8	MtBA25G04F1	[AAC]5
MTIC132	TTCCACCTTGACCACTGTTG	CCCCAAATTTACACAGATCTTCA	7	MtBC03G06R1	[AG]7
MTIC167	TGTTGTTCCAAAATTTGTCTCC	TGGAAAAGTGGGGTCTGTTT	1	MtBA07D05F1	[TC]7
MTIC186	TGGTGCTGGAAAGAAGAAGG	CACAGAGCTTCCAAAGTTGC	4	MtBA21H11F1	[AG]9
MTIC263	AATCAAAACCAATCATCACC	GTGTTCCCCAGTTCTCAGT	7	MtBB27H08F1	[CAT]6
MTIC315	CATTTCTTCATTCTTGCACA	GGCTTGAACCACAAAAGATA	2	MtBA24G01F1	[TTC]9
MTIC377	AACCCTTCCTTCCTTCCAAA	GCCATTGTTGATTGGGTTTT	3	MtBA03D10F1	[TC]6
MTIC435	TGGTGAGATAGAGGGAAGTG	ATCGAACAACAACTTTCACA	4	MtBA09A02F1	[TC]5
MTIC458	GAGTACTTCAATTCCCATGC	CACAGGATCACTCAGTAGCA	3	MtBC18H02R1	[GT]5
MTIC477	TGAGATCAGTGTGCCATAGA	TACTTCTCCTCCGGCAAA	4	MtBB33C11F1	[GAA]5
MTIC485	CTCTACACTCATTGCCCTTCTC	AAGAATGCCTATGAATGTGAAAC	4	MtBA28C04F1	[TC]10

LITERATURE CITED

GUTTIEREZ M. V., M. C. VAZ PATTO, T. HUGUET, J. I. CUBERO, M. T. MORENO *et al.*, 2005 Cross species amplification of *Medicago truncatula* microsatellites across three major pulse crops. Theoretical and Applied Genetics **110**: 1210-1217.

JULIER B., S. FLAJOULOT, P. BARRE, G. CARDINET, S. SANTONI *et al*., 2003 Construction of two genetic linkage maps in cultivated tetraploid alfalfa (*Medicago sativa*) using microsatellite and AFLP markers. BMC Plant Biology **3**: 9

LORIDON K, K. MCPHEE, J. MORIN, P. DUBREUIL, M-L. PILET-NAYEL *et al*., 2005 Microsatellite marker polymorphism and mapping in pea (*Pisum sativum* L.). Theortical and Applied Genetics **111**: 1022-1031.

MUN, J.-H., D.-J. KIM, H.-K. CHOI, J. GISH, F. DEBELLÉ *et al*., 2006 Distribution of microsatellites in the genome of *Medicago truncatula*: a resource of genetic markers that integrate genetic and physical maps. Genetics **172:** 2541-2555.

THOQUET P., M. GHÉRARDI, E. P. JOURNET, A. KERESZT, J. M. ANÉ *et al*., 2002 The molecular genetic linkage map of the model legume *Medicago truncatula*: an essential tool for comparative legume genomics and the isolation of agronomically important genes. BMC Plant Biology **2**: 1.

VOS P., R. HOGERS, M. BLEEKER, M. REIJANS, T. VAN DE LEE *et al*., 1995 AFLP : a new technique for DNA fingerprinting. Nucleic Acids Research **23**:4407-4414.
