# Supplementary material for: Mapping the Genetic Basis of Symbiotic Variation in Legume-Rhizobium Interactions in Medicago truncatula
Source: G3 (Bethesda). 2012 Nov 1;2(11):1291–303. doi: 10.1534/g3.112.003269 (PMC3484660; doi:10.1534/g3.112.003269)
Supplement: Supporting Information [file supp_2.11.1291_FileS2.pdf]

File S2

Supporting Materials and methods

Molecular marker amplification

AFLP assays were mainly conducted as described by VOS *et al*. (1995). DNA was digested by *EcoRI* plus *MseI* restriction enzymes. After ligation of double-stranded adaptors to the ends of the restriction fragments, pre-amplification was performed with *EcoRI* and *MseI* primer pairs, with one selective nucleotide at their 3'-end. The selective amplification was then performed with *EcoRI* and *MseI* primer pairs with two or three selective nucleotides at their 3'-end. The *EcoRI*+2/3 primers were labeled with either infrared dye IRD700 or IRD800 fluorochrome (MWG Biotech). Electrophoresis of the amplified products was carried out on 5.5% denaturing polyacrylamide sequencing gel and polymorphic bands were detected with a LICOR IR² automated Sequencer (LI-COR Biosciences). Image analysis of AFLP fragments was conducted using AFLP Quantar-Pro software v1.04 (Keygene, Wageningen, The Netherlands), according to the manufacturer's instructions. AFLP markers were generated following polymorphism tests using 64 selective AFLP primer pairs, from the four EcoRI-AN(N) primers used in THOQUET *et al*. (2002) and 16 MseI-CNN primers (see below, Table S6). Each AFLP marker was named using a code for the *EcoRI*/*MseI* primer combination as defined by the international nomenclature, followed by the length of the fragment (in base pairs) estimated by AFLP Quantar-Pro.

SSR amplifications were carried out using the procedure described on pea by LORIDON *et al.* (2005), with fluorescently labeled (IRD 700 or IRD 800) forward primers. A unique annealing temperature (Tm=55°C) was used for amplification of all the SSRs. Amplified products were electrophoresised on a 6.5 % denaturing polyacrylamide gel and visualized on a LICOR IR² automated Sequencer (Li-Cor Inc., Lincoln, NE, USA). Polymorphic bands were scored visually. SSR markers were derived from previous studies reporting on development of SSR markers from *M. truncatula* EST sequences (JULIER *et al.* 2003; GUTTIEREZ *et al.* 2005) or from *M. truncatula* sequenced BAC clones (MUN *et al.* 2006). Genebank EST or BAC identification number, SSR repetitive motifs and primer sequences are described, for most of the SSR markers, as electronic supplementary material in JULIER *et al*. (2003), GUTTIEREZ *et al*. (2005) and MUN *et al*. (2006). Information on 11 additional SSR markers is indicated in Table S7 (see below; HUGUET *at al*., unpublished). SSR markers from JULIER *et al.* (2003) and GUTTIEREZ *et al.* (2005) are designed "MTIC" followed by the SSR number. SSR markers from MUN *et al.* (2006) are named "MtB" followed by the SSR number.

Table S6 AFLP primer combinations used to generate fingerprints

	E-AC [a]	E-AG	E-AT	E-AGA
M-CAA [b]	E12M47	E13M47 (PB)	E14M47	E39M47
M-CAC	E12M48 (PI [c])	E13M48 (PA)	E14M48 (PD)	E39M48
M-CAG	E12M49	E13M49	E14M49 (PE)	E39M49
M-CAT	E12M50 (PK)	E13M50 (PC)	E14M50	E39M50
M-CCA	E12M51	E13M51	E14M51	E39M51 (PG)
M-CCC	E12M52	E13M52	E14M52	E39M52
M-CCG	E12M53	E13M53	E14M53	E39M53
M-CCT	E12M54	E13M54	E14M54	E39M54
M-CGA	E12M55	E13M55	E14M55	E39M55
M-CGC	E12M56	E13M56	E14M56	E39M56
M-CGG	E12M57	E13M57 (PM)	E14M57	E39M57
M-CGT	E12M58	E13M58	E14M58	E39M58 (PF)
M-CTA	E12M59	E13M59 (PL)	E14M59	E39M59
M-CTC	E12M60	E13M60	E14M60	E39M60
M-CTG	E12M61 (PH)	E13M61	E14M61	E39M61
M-CTT	E12M62 (PJ)	E13M62	E14M62	E39M62

[a] E indicates *Eco*RI primer 5’-GAC TGC GTA CCA ATT C+ANN-3’
[b] M indicates *Mse*I primer 5’-GAT GAG TCC TGA GTA A+CNN-3’
[c] In brackets, code names of *Eco*RI/*Mse*I primer combinations used in THOQUET *et al*. (2002)

Table S7 List of additional Medicago truncatula EST microsatellite primers used in this study (Huguet, unpublished). Primer sequences, linkage group in M. truncatula, GenBank EST identification and repeititve motif included.

	Primers (5'-3')		Linkage group	GenBank EST Idenitification	Repetitive motif
	Forward	Reverse			
MTIC6	CACAGTACTGCCACCGTCAA	GTCGGAGGTGAGAGGTTGAA	8	MtBA25G04F1	[AAC]5
MTIC132	TTCCACCTTGACCACTGTTG	CCCCAAATTTACACAGATCTTCA	7	MtBC03G06R1	[AG]7
MTIC167	TGTTGTTCCAAAATTTGTCTCC	TGGAAAAGTGGGGTCTGTTT	1	MtBA07D05F1	[TC]7
MTIC186	TGGTGCTGGAAAGAAGAAGG	CACAGAGCTTCCAAAGTTGC	4	MtBA21H11F1	[AG]9
MTIC263	AATCAAAACCAATCATCACC	GTGTTCCCCAGTTCTCAGT	7	MtBB27H08F1	[CAT]6
MTIC315	CATTTCTTCATTCTTGCACA	GGCTTGAACCACAAAAGATA	2	MtBA24G01F1	[TTC]9
MTIC377	AACCCTTCCTTCCTTCCAAA	GCCATTGTTGATTGGGTTTT	3	MtBA03D10F1	[TC]6
MTIC435	TGGTGAGATAGAGGGAAGTG	ATCGAACAACAACTTTCACA	4	MtBA09A02F1	[TC]5
MTIC458	GAGTACTTCAATTCCCATGC	CACAGGATCACTCAGTAGCA	3	MtBC18H02R1	[GT]5
MTIC477	TGAGATCAGTGTGCCATAGA	TACTTCTCCTCCGGCAAA	4	MtBB33C11F1	[GAA]5
MTIC485	CTCTACACTCATTGCCCTTCTC	AAGAATGCCTATGAATGTGAAAC	4	MtBA28C04F1	[TC]10

LITERATURE CITED

GUTTIEREZ M. V., M. C. VAZ PATTO, T. HUGUET, J. I. CUBERO, M. T. MORENO *et al.*, 2005 Cross species amplification of *Medicago truncatula* microsatellites across three major pulse crops. Theoretical and Applied Genetics **110**: 1210-1217.

JULIER B., S. FLAJOULOT, P. BARRE, G. CARDINET, S. SANTONI *et al*., 2003 Construction of two genetic linkage maps in cultivated tetraploid alfalfa (*Medicago sativa*) using microsatellite and AFLP markers. BMC Plant Biology **3**: 9

LORIDON K, K. MCPHEE, J. MORIN, P. DUBREUIL, M-L. PILET-NAYEL *et al*., 2005 Microsatellite marker polymorphism and mapping in pea (*Pisum sativum* L.). Theortical and Applied Genetics **111**: 1022-1031.

MUN, J.-H., D.-J. KIM, H.-K. CHOI, J. GISH, F. DEBELLÉ *et al*., 2006 Distribution of microsatellites in the genome of *Medicago truncatula*: a resource of genetic markers that integrate genetic and physical maps. Genetics **172:** 2541-2555.

THOQUET P., M. GHÉRARDI, E. P. JOURNET, A. KERESZT, J. M. ANÉ *et al*., 2002 The molecular genetic linkage map of the model legume *Medicago truncatula*: an essential tool for comparative legume genomics and the isolation of agronomically important genes. BMC Plant Biology **2**: 1.

VOS P., R. HOGERS, M. BLEEKER, M. REIJANS, T. VAN DE LEE *et al*., 1995 AFLP : a new technique for DNA fingerprinting. Nucleic Acids Research **23**:4407-4414.
